# Target Functionalized Carbon Dot Nanozymes with Dual‐Model Photoacoustic and Fluorescence Imaging for Visual Therapy in Atherosclerosis

**DOI:** 10.1002/advs.202307441

**Published:** 2023-12-25

**Authors:** Qiao Chen, Xinmei Duan, Yao Yu, Rongrong Ni, Guojing Song, Xu Yang, Li Zhu, Yuan Zhong, Kun Zhang, Kai Qu, Xian Qin, Wei Wu

**Affiliations:** ^1^ Key Laboratory for Biorheological Science and Technology of Ministry of Education State and Local Joint Engineering Laboratory for Vascular Implants Bioengineering College of Chongqing University Chongqing 400044 China; ^2^ Thyroid Breast Surgery Department Dazhou Central Hospital Dazhou 635000 China; ^3^ Medical Department Southwest Hospital Third Military Medical University Chongqing 400038 China; ^4^ Urology Southwest Hospital Third Military Medical University Chongqing 400038 China; ^5^ Chongqing University Three Gorges Hospital Chongqing 404000 China; ^6^ Jin Feng Laboratory Chongqing 401329 China

**Keywords:** atherosclerosis, carbon dots, fluorescence imaging, photoacoustic imaging, superoxide dismutase nanozyme

## Abstract

Multifunctional nanomedicines have been used in atherosclerosis theranostics. Herein, phosphatidylserine‐specific peptide CLIKKPF‐functionalized carbon‐dots nanozymes (pep‐CDs) are reported for specific and efficient noninvasive theranostic of atherosclerosis. Surprisingly, pep‐CDs are discovered to not only inherit the inherent properties of carbon dots (CDs), including deep‐red fluorescence emission, photoacoustic response, and superoxide dismutase‐like antioxidant, and anti‐inflammatory activities but also possess the ability to target recognition on foam cells and target localization on plaques due to the specific interaction of CLIKKPF with phosphatidylserine on the membrane outer surface of foam cells. Furthermore, the target localization effect of pep‐CDs vastly promotes the efficient accumulation of CDs in plaque, thus maximizing AS theranostic of CDs. Interestingly, pep‐CDs could be developed to image plaque for monitoring atherosclerosis pathological progression in real‐time resulting from the different content of foam cells. This work on the one hand proposes a simple and feasible strategy to construct theranostic nanoplatform employing only a single functional unit (i.e., multifunctional CDs) to simplify the fabrication procedure, on the other hand, highlights the advantages of the active target auxiliary mode for atherosclerosis theranostic applications.

## Graphical abstract

A multifunctional carbon dot nanozyme is developed based on phosphatidylserine (PS)‐specific peptide CLIKKPF‐functionalized carbon dots (pep‐CDs). This study highlights a simple and feasible strategy to construct theranostic nanoplatform employing only a single functional unit (i.e., multifunctional CDs) and provides a promising paradigm for highly specific and efficient noninvasive theranostic of atherosclerosis (AS).

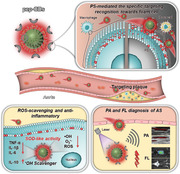

## 1 Introduction

Atherosclerosis (AS), a chronic progressive inflammatory disease with multifocal characteristics, is the common pathological basis of a variety of cardiovascular diseases, encompassing ischemic heart disease, coronary artery disease, and stroke.^[^ ^1^, ^2^, ^3^ ^]^ In the early stages of AS formation, early management is much more important and effective. However, it is a great challenge to accurately diagnose due to the non‐obvious symptoms. In the later stage of AS progression, blood flow limitation or obstruction caused by advanced vulnerable plaques will lead to a series of serious cardiovascular diseases, thus posing a great threat to human life.^[^ ^4^, ^5^ ^]^ Hence, early precise diagnosis and effective treatment of AS are highly appreciated to reduce the incidence of cardiovascular disease. Benefiting from the booming development of nano‐biomedicine, nanoparticles integrating imaging and therapeutic properties are defined as “theranostic agents” to implement precise therapy on the basis of dynamic monitoring of AS plaques.^[^ ^6^, ^7^ ^]^ However, to construct these multifunctional theranostic agents, it is usually necessary to combine multiple functional units, which inevitably requires a complex preparation process. Additionally, the potential toxicity, low accumulation efficiency in plaques, and inconspicuous therapeutic effect of multifunctional theranostic agents also greatly hinder their further development for clinical application. Therefore, it is still very anticipated to prepare multifunctional theranostic agents with satisfactory AS diagnosis and treatment function through a simple and feasible method.

CDs have shown great potential for applications in sensing, bioimaging, and disease diagnosis due to their favorable properties such as tunable optical properties, excellent biocompatibility, easy surface functionalization, low cost, and particularly the potential phototriggered imaging functions including photoacoustic (PA) and fluorescence (FL) imaging.^[^ ^8^, ^9^, ^10^, ^11^ ^]^ Most intriguingly, the inherent advantages of certain specially designed CDs enable them to meet the crucial requirements for diagnosis and treatment of oxidative stress‐related diseases, for instance, deep‐red FL emission properties, antioxidant, anti‐inflammatory, and importantly enzyme‐like activities.^[^ ^12^, ^13^, ^14^ ^]^ Therefore, CDs could be exploited as a single functional component to construct AS theranostic nanoplatform, significantly simplifying the sophisticated fabrication process and the complex property regulation. Nevertheless, non‐specific distribution and poor bioavailability usually limit their competent accumulation at the lesion site, thus diminishing their diagnostic performance.^[^ ^15^ ^]^ In order to boost the application value of CDs in AS theranostic, one of the key breakthrough points to be resolved is to appropriately increase their target cumulative efficiency without affecting their inherent performances and functions.

Given the highly complex microenvironment of AS plaques, involving a variety of cells (such as foam cells and endothelial cells), molecules, lipids, enzymes, weak acidity, and rich reactive oxygen species (ROS), the proposed passive targeting (targeting the natural characteristics of plaques) and active targeting strategies provide a favorable solving idea to eliminate the above‐mentioned problems.^[^ ^16^, ^17^ ^]^ Compared with the passive targeting strategy, the active targeting strategy is highly attractive because of their enhanced targeting delivery efficiency to lesion and most importantly the capacity to actively search their “target.”^[^ ^18^ ^]^ Since foam cells play a considerable role in the occurrence and development of AS, they are widely regarded as a promising target in AS theranostics.^[^ ^19^, ^20^, ^21^ ^]^ Therefore, it is speculated that the binding of versatile CDs and suitable foam cell‐related ligands may relieve the contradiction between the enhanced accumulation at plaque lesion and the rapid clearance in vivo.

**Scheme 1:**
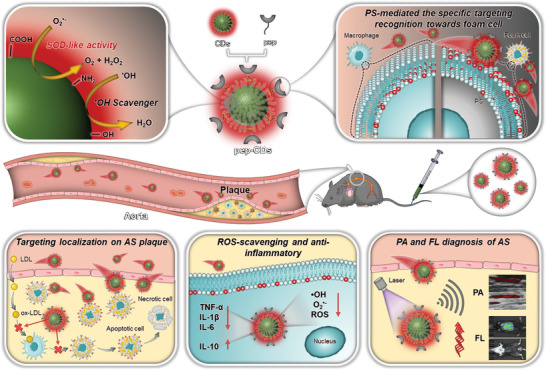
Schematic diagram of pep‐CDs with PS targeting function for AS theranostic.

Phosphatidylserine (PS), a representative molecule in the membrane outer surface of foam cells, has been demonstrated to interact specifically with peptide CLIKKPF.^[^ ^22^, ^23^, ^24^ ^]^ Herein, multifunctional nanoparticles (termed pep‐CDs) with the enhanced plaque accumulation were fabricated by facile coupling of peptide CLIKKPF and CDs for specific target AS theranostic (**Scheme** **1**). pep‐CDs are endowed with several prominent features: i) the inherent properties of CDs, such as deep‐red FL emission, sensitive PA response, and SOD‐like antioxidant, and anti‐inflammatory activities, enable them to perform imaging and therapeutic functions through a single component, thereby avoiding sophisticated preparation processes; ii) the introduced PS targeting peptide CLIKKPF not only promotes the specific target recognition of CDs on foam cells, but also enhances the target accumulation of CDs on AS plaque, which lays the foundation for the visual treatment of CDs; iii) compared with the free CDs, pep‐CDs in plaque lesion maximizes the ROS elimination and anti‐inflammatory effects because of the accessible therapeutic window mediated by their enhanced accumulation. Thus, this work proposed a simple and feasible strategy to construct a target nanoplatform for FL/PA imaging guided visual therapy of AS.

## 2 Results and Discussion

### 2.1 Synthesis and Characterizations of pep‐CDs Nanozyme

**Figure 1:**
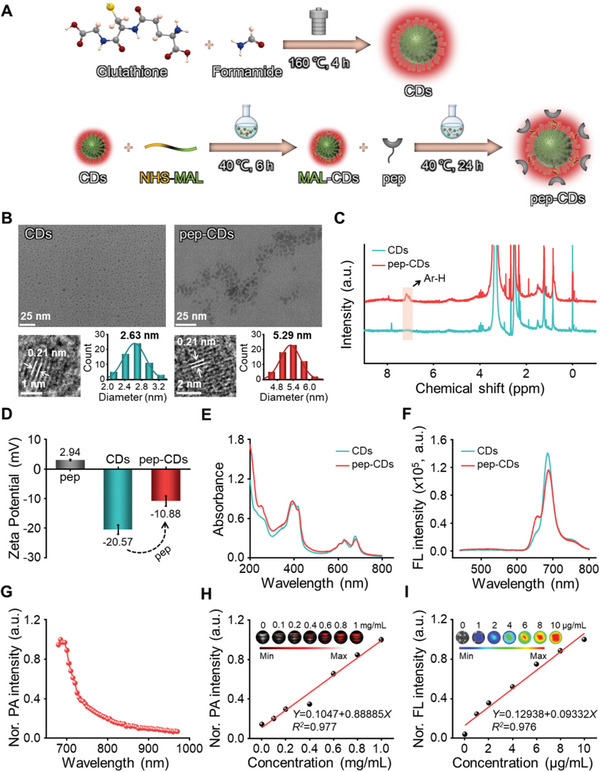
Synthesis and characterizations of pep‐CDs. A) Synthesis diagram of CDs and pep‐CDs. B) TEM images, HRTEM images, and size distribution histograms of CDs and pep‐CDs. C) ^1^H NMR spectra of CDs and pep‐CDs. D) Zeta potential of pep, CDs, and pep‐CDs. E) UV–Vis absorption spectrum and F) FL emission spectra (*λ*_ex_ = 420 nm) of CDs and pep‐CDs. G) PA signal intensity of pep‐CDs (1 mg mL^‐1^) at different excitation wavelengths. H) In vitro PA images and signal intensity (λ_ex_ = 685 nm) of pep‐CDs at different concentrations. I) In vitro FL images and signal intensity of pep‐CDs at different concentrations. Data in (D,G) were illustrated as mean ± s.d. (*n* = 3).

Deep‐red‐emitting CDs with enzyme‐like activity were synthesized by the one‐step solvothermal method from glutathione and formamide according to previously reported methods with slight modifications (**Figure** **1**A).^[^ ^25^, ^26^ ^]^ In order to imbue CDs with the target localization function, a heterobifunctional crosslinker 3‐maleimidopropionic acid *N*‐hydroxysuccinimide ester was used to functionalize CDs with PS‐specific peptide CLIKKPF, thus fabricating carbon‐dots nanoenzymes with “localization” function, namely pep‐CDs. First, the successful preparation of pep‐CDs was studied by transmission electron microscopy (TEM). As shown in Figure 1B, free CDs and pep‐CDs were presented in a well‐dispersed quasi‐spherical morphology with the average diameters of 2.63 nm and 5.29 nm, respectively. In addition, high‐resolution TEM (HRTEM) images were observed that both of them contain identical lattice fringes with a lattice space of 0.21 nm, indicating the presence of graphite‐like structures. The hydrodynamic diameters of CDs and pep‐CDs were about 2.44 and 6.76 nm (Figure S1, Supporting Information). Subsequently, the composition and surface structure of materials were analyzed by X‐ray photoelectron spectroscopy (XPS), Fourier transform infrared (FT‐IR) and ^1^H nuclear magnetic resonance (^1^H NMR) spectra. The XPS spectra of CDs were shown in Figure S2A (Supporting Information). Four peaks at 400, 285, 531, and 163 eV were observed according to N 1s, C 1s, O 1s, and S 2p, respectively, manifesting CDs mainly containing N, C, O, and S elements with molar ratio of 22.95: 50.73: 25.64: 0.68. Additionally, the high‐resolution (HR) XPS spectra of N 1s, C 1s, O 1s, and S 2p were fitted (Figure S2B–E, Supporting Information). In the HR N 1s spectra, the binding energies at 399.0, 399.9, and 400.5 eV were attributed to amino N, pyrrolic N, and graphitic N, respectively, demonstrating the presence of amino groups and nitrogen‐containing heterocyclic rings. In HR C 1s spectra, the binding energies at 284.5, 285.1, 285.9, 287.9, and 289.0 eV were assigned to the C═C/C─C, C─S, C─N, C═N/C═O, and N─C═O bonds, respectively. In HR O 1s spectra, the binding energies at 531.1 and 532.4 eV were attributed to C═O and C─OH/C─O─C. In HR S 2p spectra, the binding energies at 161.8, 163.3, 164.9, 168.0 eV were ascribed to thiolate, 2p_3/2_ and 2p_1/2_ of thiophene S, and oxidized S, respectively. The ^1^H NMR spectra of CDs and pep‐CDs were presented in Figure 1C. In contrast with CDs, ^1^H NMR spectra of pep‐CDs exhibited an additional shoulder peak from 7.04 to 7.21 ppm ascribing to the aromatic ring of phenylalanine presented in the peptide CLIKKPF, confirming the successful coupling of peptide CLIKKPF on the CDs. Furthermore, in the FT‐IR spectra (Figure S3, Supporting Information), the C═O and ─NH─ peaks attributed to the amide bond of CDs were found at 1667 and 1614 cm^−1^, respectively. After modifying peptide CLIKKPF, the characteristic peaks of C═O and ─NH─ were slightly shifted to 1665 and 1633 cm^−1^, respectively, which was mainly related to the characteristic structure of the peptide, further confirming the successful preparation of pep‐CDs. After coupling, the Zeta potential positively shifted from −20.57 mV of free CDs to −10.88 mV of pep‐CDs due to the introduction of peptides (Figure 1D).

To further investigate the optical properties of pep‐CDs, the ultraviolet–visible (UV–Vis) absorption and FL emission/excitation spectra were characterized. As illustrated in Figure 1E, CDs and pep‐CDs revealed the similar absorption behavior with several strong absorption bands at 350–450 and 550–750 nm at the same concentration, and wide absorption bands trailing into the near‐infrared (NIR) region, proving their potential for NIR light‐triggered PA imaging. Based on the concentration‐dependent absorption spectra of CDs, calibration curves, and typical absorption spectra of pep‐CDs exhibited in Figure S4 (Supporting Information), the peptide loading efficiency on CDs was determined to be 42.3% (mass percentage). Moreover, as illustrated in Tables S1 and S2 and Figure S5 (Supporting Information), the high‐performance liquid chromatography‐tandem mass spectrometry (HPLC‐MS/MS) method proved that only a very small amount of free peptides (2.59%) was present in the pep‐CDs. The concentrations associated with pep‐CDs in the subsequent manuscript were expressed as actual concentrations of CDs. As depicted in Figure 1F, both CDs and pep‐CDs possessed the deep‐red emission properties (*λ*_ex_ = 420 nm), and their optimal emission was located at about 684 nm. Surprisingly, the optimal emission intensity of pep‐CDs was only slightly reduced by about 20% compared with an equivalent amount of the free CDs, indicating the occurrence of a mild electron/energy transfer process after coupling. Additionally, pep‐CDs displayed the similar FL excitation spectra to CDs, also suggesting the perfectly retained optical properties from CDs (Figure S6, Supporting Information). Subsequently, the dispersibility and stability of pep‐CDs were reflected by FL variation and aggregation of pep‐CDs dispersion in different biological solvents (H_2_O, PBS, and DMEM). As shown in Figure S7 (Supporting Information), only slight FL alterations and no obvious precipitation were found from pep‐CDs dispersion in H_2_O, PBS, and DMEM during the whole observation period from 0 to 6 d, indicating the favorable dispersibility and stability of pep‐CDs. These results demonstrated the potential of pep‐CDs for sensitive FL bioimaging.

Finally, considering the wide absorption band in the NIR region and deep‐red FL emission, the PA and FL response abilities of pep‐CDs were investigated. The PA signal of pep‐CDs at the broad excitation wavelength of 680–970 nm was observed as Figure 1G, where the strongest PA signal was obtained at the excitation wavelength of 685 nm. Therefore, 685 nm was selected as the optimal excitation wavelength for subsequent PA imaging‐related experiments. Expectedly, the corresponding PA signal intensity gradually strengthened as pep‐CDs concentrations increased from 0 to 1 mg mL^‐1^ and was linear with pep‐CDs concentration (Figure 1H), demonstrating their potential for deep tissue PA imaging. In addition, FL signal intensity also linearly enhanced with the raising of pep‐CDs concentration (Figure 1I). Given the superior optical properties and sensitive PA/FL response, pep‐CDs could be considered a promising candidate for the advanced multimodal imaging.

### 2.2 Antioxidant, ROS Scavenging, and SOD‐Like Activities of pep‐CDs Nanozyme

**Figure 2:**
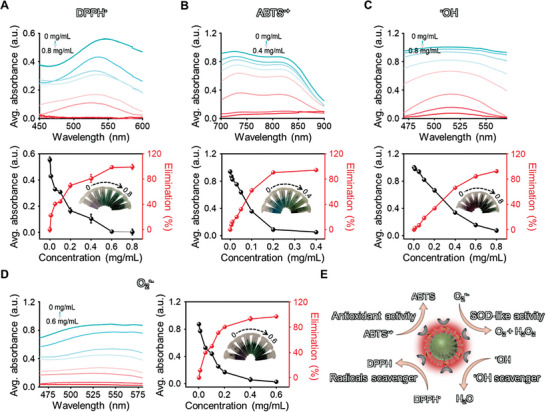
Antioxidant, ROS scavenging, and SOD‐like activities of pep‐CDs nanozyme. A–D) UV–vis absorption spectra of DPPH^•^, ABTS^•+^, ^•^OH, and O_2_^•−^ after incubation with different concentrations of pep‐CDs, and the corresponding absorbance of each system and the elimination efficiency of DPPH^•^, ABTS^•+^, ^•^OH, and O_2_^•−^ by pep‐CDs. E) Schematic diagram of antioxidant, ROS scavenging, and SOD‐like of pep‐CDs nanozyme. Data in (A–D) were illustrated as mean ± s.d. (*n* = 3).

Since antioxidant activity generally determines antioxidant stress capacity, the total antioxidant activity of pep‐CDs was evaluated by using 1,1‐diphenyl‐2‐picrylhydrazyl radical (DPPH^•^) as an indicator. As illustrated in **Figures** **2**A and S8 (Supporting Information), with the gradual increased concentration of CDs and pep‐CDs especially in the low concentration range, the removal efficiency of DPPH^•^ sharply promoted, which might be related to the abundant reducing functional groups on the CDs surface. CDs and pep‐CDs also showed excellent ABTS^•+^ radicals scavenging activity (Figures 2B and S9, Supporting Information). Hydroxyl radicals (^•^OH) are considered to be the most toxic ROS due to their high reduction potential.^[^ ^27^ ^]^ Interestingly, the capacity of CDs and pep‐CDs to scavenge ^•^OH was also discovered. As shown in Figures 2C and S10 (Supporting Information), with the increased concentration of CDs and pep‐CDs, ^•^OH signal intensity significantly decreased, and ^•^OH scavenging efficiency obviously accelerated. The SOD‐like activity of pep‐CDs was assessed through employing nitrotetrazolium blue chloride (NBT) as the O_2_^•−^ indicator. The absorption value of NBT could be specifically decreased by O_2_^•−^. As illustrated in Figures 2D and S11 (Supporting Information), with the increased concentration of CDs, the inhibition rate of CDs and pep‐CDs on O_2_^•−^ generation gradually enhanced. Surprisingly, the inhibition rate of O_2_^•−^ generation was close to 100% when the concentration reached 0.6 mg mL^‐1^, indicating their favorable SOD mimicking enzyme activity. Taken together, pep‐CDs nanozyme inherited the excellent antioxidant, ROS scavenging, and SOD‐like activities of CDs (Figure 2E), being regarded as a general antioxidant nanozyme for the oxidative stress‐related diseases therapy.

### 2.3 In Vitro PS‐Mediated the Specific Targeting Recognition Ability toward Foam Cells

Prior to in vitro biological evaluation, the cytotoxicity of CDs and pep‐CDs in cells associated with AS, including macrophages, endothelial cells, and vascular smooth muscle cells (VSMC), was first determined via MTS assay. As shown in Figure S12 (Supporting Information), relatively high cell viability ratios were detected in mouse mononuclear macrophage cell line (RAW264.7), human umbilical vein endothelial cell line (HUVEC), and mouse aortic smooth muscle cell line (MOVAS) after incubation with CDs and pep‐CDs at different concentration for 12 or 24 h. Even at the concentrations up to 400 µg mL^‐1^, the percentage of living cells in the three type cells was still above 90%. Therefore, these results indicated the low cytotoxicity and excellent biocompatibility of CDs and pep‐CDs.

**Figure 3:**
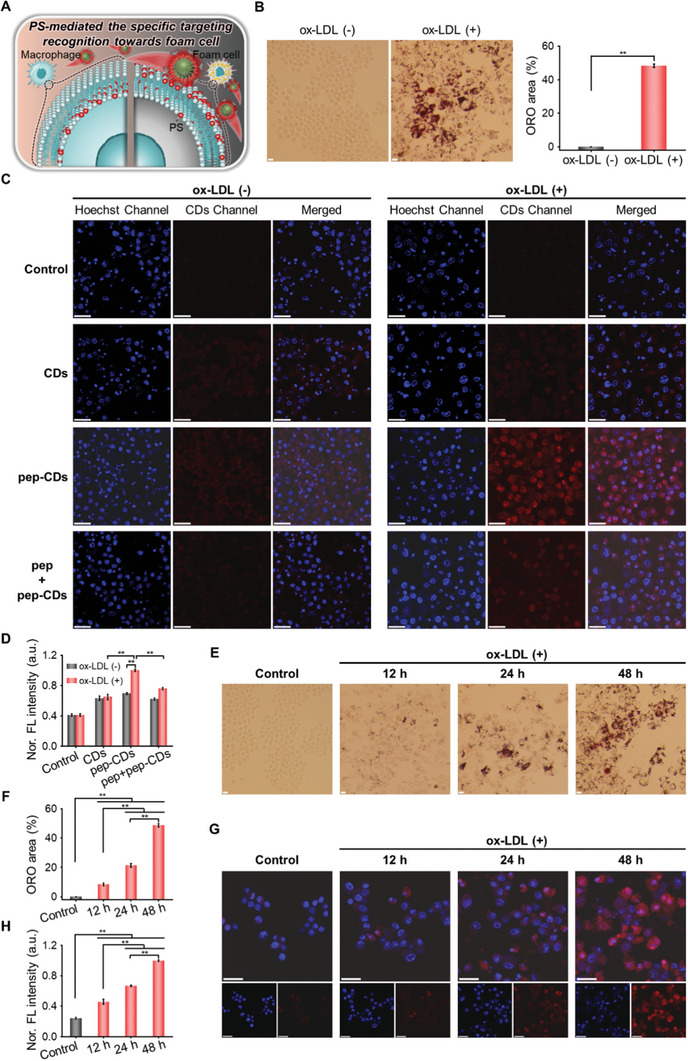
In vitro PS‐mediated the specific targeting recognition ability towards foam cells of pep‐CDs. A) Schematic diagram of PS‐mediated the specific targeting recognition towards foam cells of pep‐CDs. B) ORO staining images and corresponding ORO areas of RAW264.7 macrophages after incubation with or without ox‐LDL for 48 h (scale bar: 50 µm). C) CLSM images and D) corresponding quantitative analysis of FL signal intensities of RAW264.7 macrophages pre‐incubated with or without ox‐LDL for 48 h followed by treatments of PBS (Control), CDs, pep‐CDs, and pep+pep‐CDs, respectively (scale bar: 25 µm). E) ORO staining images and F) corresponding ORO areas of RAW264.7 macrophages incubated with or without ox‐LDL for 12, 24, and 48 h (scale bar: 50 µm). G) CLSM images and H) corresponding quantitative analysis of FL signal intensities of RAW264.7 macrophages incubated with or without ox‐LDL for 12, 24, and 48 h followed by treatments of pep‐CDs (scale bar: 25 µm). Data in (B, D, F, H) were illustrated as mean ± s.d. (*n* = 3). **p < 0.01.

In view of the proposed idea that pep‐CDs specifically targets foam cells through PS functional modification (**Figure** **3**A). Subsequently, the targeting recognition towards foam cells of pep‐CDs was verified via binding affinity experiments in vitro. First, RAW264.7 macrophages were selected to form foam cells by oxidized‐low density lipoprotein (ox‐LDL) stimulation. The results of oil red O (ORO) staining displayed that the lipid content in ox‐LDL‐stimulated macrophages was significantly increased compared with the non‐ox‐LDL‐stimulated macrophages (Figure 3B), indicating the successful construction of the foam cell model. Next, in vitro binding affinity was observed by confocal laser scanning microscope (CLSM). As revealed in Figure 3C,D, for foam cells overexposed to PS, pep‐CDs group showed significantly stronger red fluorescence, while CDs and blocked group only detected the weak fluorescence due to the absence or blocking of PS‐specific peptide, suggesting the enhanced uptake of pep‐CDs through the specific interaction between the PS exposed on the surface of foam cells and the PS‐specific peptide CLIKKPF on the surface of CDs. At the same time, for unstimulated macrophages, no remarkable red fluorescence was detected in CDs or pep‐CDs or blocking groups. This result was interesting because it revealed that pep‐CDs could selectively distinguish between the normal macrophages and the macrophage‐derived foam cells, consequently enhancing their specific targeting. In addition, the uptake of pep‐CDs by foam cells increased with the extension of the incubation time (Figure S13, Supporting Information). These results fully confirmed the outstanding targeting recognition ability toward foam cells of pep‐CDs.

Finally, the cellular uptake of pep‐CDs by cells with different degrees of foaming was investigated. First, in order to form cells with various degrees of foaming, RAW264.7 macrophages were stimulated by ox‐LDL for various times. As illustrated in Figure 3E,F, significantly more lipid content was observed with prolonged ox‐LDL stimulation, indicating successful construction of cell models with different degrees of foaming. Subsequently, the uptake of pep‐CDs by cells with various degrees of foaming was observed by CLSM. As depicted in Figure 3G,H, a stronger red fluorescence was significantly observed in the prolonged ox‐LDL stimulation group, which might be attributed to the increased PS exposure in severely foamy cells. Taken together, the excellent targeting recognition ability towards foam cells of pep‐CDs was able to enhance the specific targeting localization on AS plaques.

### 2.4 In Vitro ROS‐Scavenging Activity of pep‐CDs Nanozyme in Macrophages

**Figure 4:**
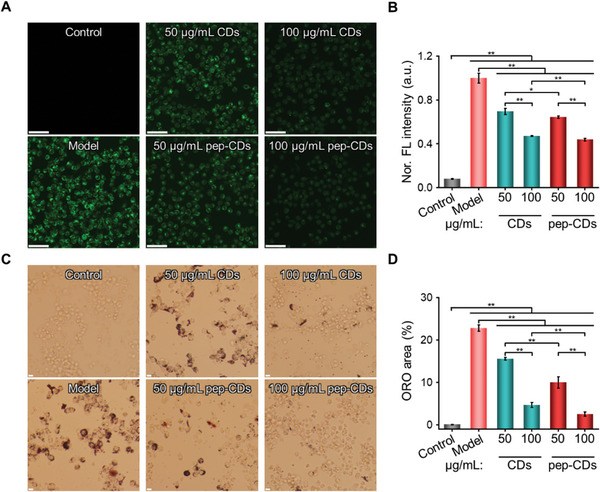
In vitro therapeutic effect of pep‐CDs nanozyme. A) ROS staining CLSM images (fluorescent probe: DCFH‐DA) and B) corresponding quantitative analysis of FL signal intensities of RAW264.7 macrophages pre‐incubated with PBS (Control and Model), CDs, and pep‐CDs for 4 h, respectively, and then treated with or without lipopolysaccharide (LPS) for 1.5 h (scale bar: 25 µm). C) ORO staining images and D) corresponding ORO areas of RAW264.7 macrophages pre‐incubated with PBS (Control and Model), CDs, and pep‐CDs for 6 h, respectively, and then treated with or without ox‐LDL for 24 h (scale bar: 50 µm). Data in (B, D) were illustrated as mean ± s.d. (*n* = 3). *p < 0.05, **p < 0.01.

In AS pathology, the uncontrolled excess ROS results in oxidative stress, subsequently causing cell and tissue damage and subsequent inflammation and amplification of oxidative stress.^[^ ^28^ ^]^ Encouraged by the favorable antioxidant, ROS scavenging, and SOD‐like activities of pep‐CDs, they were proposed as a potential candidate for antioxidative therapy of AS. The flow cytometry results showed that CDs and pep‐CDs could be effectively endocytosed by RAW264.7 macrophages ( Figure S14, Supporting Information), which was beneficial for the subsequent treatment. The ROS scavenging efficacy of pep‐CDs was investigated by employing 2,7‐dichlorofluorescin diacetate as an FL indicator. As shown in **Figure** **4**A,B, the cells in the model group displayed quite high levels of ROS (green fluorescence). On the contrary, the fluorescence signal of CDs or pep‐CDs‐treated groups with different formulations was significantly weakened, indicating that pep‐CDs could effectively scavenge ROS in living cells and protect cells from oxidative stress damage.

### 2.5 In Vitro Inhibiting Formation of Foam Cell

Macrophage foam cell formation is a major hallmark of AS lesions.^[^ ^29^ ^]^ Therefore, the inhibitory effects of CDs and pep‐CDs on foam cell formation were examined by ORO staining. As illustrated in Figure 4C,D, RAW264.7 macrophages treated with 80 µg mL^‐1^ ox‐LDL for 24 h revealed a significant amount of intracellular lipid droplets and the remarkable macrophage foam cell formation. Moreover, treatment with the different doses of CDs or pep‐CDs notably decreased intracellular lipid droplets and foam cell formation, verifying that pep‐CDs could significantly attenuate formation of the macrophage‐derived foam cell.

### 2.6 In Vivo Targeting Localization and PA/FL Imaging toward AS Plaques

**Figure 5:**
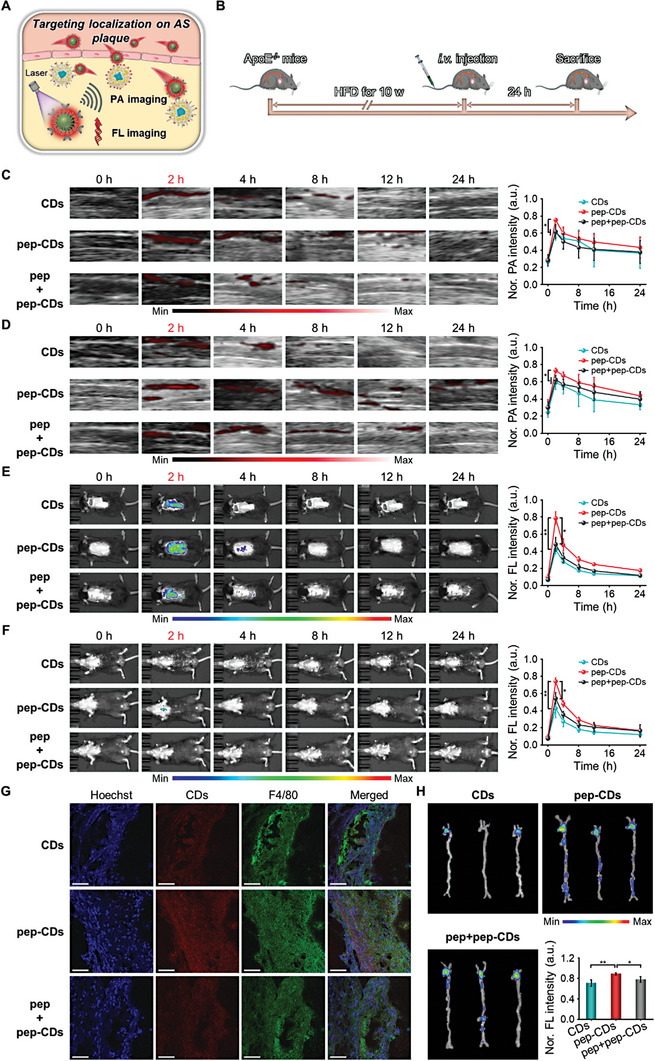
In vivo targeting localization and PA/FL imaging ability toward AS plaques of pep‐CDs. A) Schematic diagram of targeting localization and PA/FL imaging towards AS plaques of pep‐CDs. B) Schematic diagram of AS plaque imaging protocol. PA images and corresponding quantitative analysis of PA signal intensities of AS plaques in C) right and D) left carotid arteries of ApoE^−/−^ mice fed on HFD for 10 w at various time points after i.v. injection of CDs, pep‐CDs, and pep+pep‐CDs, respectively. FL images and corresponding quantitative analysis of FL signal intensities of AS plaques in typical E) prone and F) supine positions of ApoE^−/−^ mice fed on HFD for 10 w at various time points after i.v. injection of CDs, pep‐CDs, and pep+pep‐CDs, respectively. G) CLSM images of F4/80 immunofluorescence stained‐aortic root cryosections from ApoE^−/−^ mice fed on HFD for 10 w at 2 h post pep‐CDs i.v. injection (scale bar: 50 µm). H) *Ex vivo* FL images and corresponding quantitative analysis of FL signal intensities of aortas from ApoE^−/−^ mice fed on HFD for 10 w at 2 h post pep‐CDs i.v. injection. Data in (C–F, H) were illustrated as mean ± s.d. (*n* = 4–5). *p < 0.05, **p < 0.01.

Foam cells are important components of AS plaques.^[^ ^30^ ^]^ Motivated by the satisfactory extracellular PA response, optical properties, and specific targeting ability towards foam cells at the cellular level of pep‐CDs, their targeting localization and PA/FL imaging ability towards AS plaque tissue in vivo were further studied (**Figure** **5**A). First, the blood compatibility of pep‐CDs was assessed by hemolysis tests to determine their safety in vivo application. As presented in Figure S15 (Supporting Information), compared with the positive control group, negligible ruptured red blood cells and hemolysis rates below an acceptable level of 5% were observed in the supernatant of the samples treated with different concentrations of pep‐CDs, confirming the exceptional hemocompatibility of pep‐CDs.

Afterwards, the male apolipoprotein E‐deficient (ApoE^−/−^) mice were fed high fat diets (HFD) for 10 w to establish AS mouse models (Figure 5B), which were then used to explore the specific targeting accumulation and PA imaging ability of pep‐CDs on AS plaque tissue through the VEVO LAZR‐X photoacoustic imaging system. As depicted in Figure 5C, intense PA signal was detected from the right carotid plaque in the pep‐CDs‐treated group at 2 h after injection and maintained at a detectable level even after injection for 24 h, proving the superior PA imaging ability and effective accumulation of pep‐CDs at the plaque lesion. Noteworthily, in contrast to the pep‐CDs‐treated group, the PA signal intensity at plaque was notably weakened in the CDs‐ and blocker‐treated groups at 2 h after injection attributed to the deletion or blocking of PS‐specific peptides, validating the specific targeting ability of pep‐CDs on plaque tissue. As expected, the stronger PA signals from left carotid plaque in the pep‐CDs‐treated group at 2 h post‐injection also were exhibited than those in the CDs‐ and blocker‐treated groups (Figure 5D). In addition, the target FL imaging capability of pep‐CDs on AS plaque tissue was verified by the IVIS optical imaging system. As shown in Figure 5E, the peak FL signal from the aortic plaque lesion in the pep‐CDs‐treated group at 2 h after injection was captured and stronger than those in the CDs‐ and blocker‐treated groups, which was consistent with the above PA imaging phenomenon. Likewise, similar FL phenomena were observed at the aortic arch plaque lesion (Figure 5F), validating the excellent target FL imaging ability of pep‐CDs and determining the optimal time point for FL imaging of isolated aorta (i.e., 2 h post‐injection). These results effectively confirmed the targeting localization ability of pep‐CDs to AS plaques in vivo, which fully demonstrated their promising potential for the non‐invasive diagnosis of AS in vivo.

In order to further explain the potential reasons for targeting recognition towards plaque of pep‐CDs, F4/80 (macrophage foam cell markers) immunofluorescence imaging of the aortic root and FL imaging of isolated aorta were tested. As displayed in Figure 5G, under the condition of almost the equal amount of F4/80, red emission from the plaque lesion in the pep‐CDs‐treated group were quite bright, and most of them were colocalized with F4/80, while CDs‐ and blocker‐treated groups were the opposite, indicating that the interaction between PS‐specific peptides and foam cells was conducive to the accumulation of pep‐CDs at the plaque lesion. Therefore, these results confirmed that PS‐specific peptides were indispensable in the diagnosis of AS by CDs. Equally, the accumulation degree of CDs from the aortic plaque lesion in the pep‐CDs‐treated group was quite obvious and significantly higher than that in the CDs‐ and blocker‐treated groups (Figure 5H). In addition, the accumulation of CDs in healthy aortas was negligible (Figure S16, Supporting Information). In general, pep‐CDs could target the recognition of AS plaques by interacting with foam cells to enhance their accumulation.

In addition, the pharmacokinetic behavior in vivo and body distribution of pep‐CDs were evaluated. As revealed in Figure S17 (Supporting Information), pep‐CDs were almost cleared from the blood at 24 h post‐injection. As illustrated in Figure S18 (Supporting Information), pep‐CDs were mainly distributed in the liver at 2 h after injection and primarily accumulated in the kidney after 8 h injection, indicating that pep‐CDs could be mainly metabolized by the liver in the early stage and the kidney in the later stage.

### 2.7 In Vivo Real‐Time Monitoring the Pathological Progression Of AS

**Figure 6:**
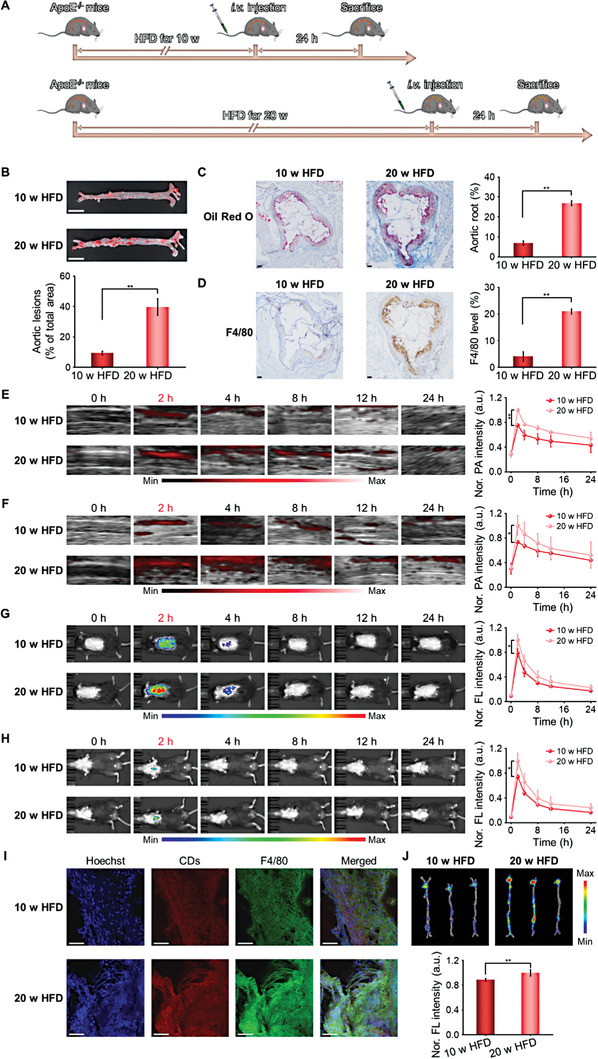
In vivo monitoring the pathological progression of AS by pep‐CDs. A) Schematic diagram of imaging protocol for AS plaque in different pathological progression. B) En face ORO staining images and corresponding quantitative analysis of the lesion area of aortas from ApoE^−/−^ mice fed on HFD for 10 and 20 w (scale bar: 5 mm). C) ORO staining images and corresponding quantitative analysis of the plaque area of aortic root cryosections from ApoE^−/−^ mice fed on HFD for 10 and 20 w (scale bar: 100 µm). D) F4/80 immunohistochemistry staining images and corresponding quantitative analysis of the plaque F4/80 expression levels of aortic root cryosections from ApoE^−/−^ mice fed on HFD for 10 and 20 w (scale bar: 100 µm). PA images and corresponding quantitative analysis of PA signal intensities of AS plaques in E) right and F) left carotid arteries of ApoE^−/−^ mice fed on HFD for 10 and 20 w at various time points after i.v. injection of pep‐CDs. FL images and corresponding quantitative analysis of FL signal intensities of atherosclerosis plaques in typical G) prone and H) supine positions of ApoE^−/−^ mice fed on HFD for 10 and 20 w at various time points after i.v. injection of pep‐CDs. I) CLSM images of F4/80 immunofluorescence‐stained aortic root cryosections from ApoE^−/−^ mice fed on HFD for 10 and 20 w at 2 h post pep‐CDs i.v. injection (scale bar: 50 µm). J) *Ex vivo* FL images and corresponding quantitative analysis of FL signal intensities of aortas from ApoE^−/−^ mice fed on HFD for 10 and 20 w at 2 h post pep‐CDs i.v. injection. Data in (B‐H, J) were illustrated as mean ± s.d. (*n* = 4–5). *p < 0.05, **p < 0.01.

Based on the favorable targeting ability of pep‐CDs to plaque in vivo, the real‐time monitoring performance of pep‐CDs on AS plaque progression in vivo was further explored. Firstly, ApoE^−/−^ mice were fed HFD for 10 and 20 w to establish mouse models of AS with different pathological progression (**Figure** **6**A). Then, the successful construction of AS models with various pathological progression was determined by ORO staining of the aorta and ORO and F4/80 immunohistochemical staining of the aortic root cryosections. As presented in Figure 6B, the larger and more ORO‐positive regions were shown in the aorta of 20 w HFD‐induced mice. In contrast, the smaller and fewer ORO‐stained areas were observed in the aorta of 10 w HFD‐induced mice. As expected, the results of ORO staining on the aortic root cryosections were consistent with the above results (Figure 6C), illustrating that the lipid core gradually enlarged and increased with prolonged HFD feeding. As depicted in Figure 6D, compared with aortic root cryosections of 10 w HFD‐induced mice, the expression of F4/80 was more plentiful in aortic root cryosections of 20 w HFD‐induced mice, suggesting that the number of macrophage foam cells gradually elevated with the extension of HFD feeding time. Taken together, the successful construction of AS models with different pathological progression was clearly confirmed, which was beneficial to the conduct of subsequent animal imaging experiments.

Subsequently, the monitoring effect of pep‐CDs on the various pathological progression of AS plaque in vivo was reflected by PA and FL imaging. As displayed in Figure 6E,F, the strong PA signal was detected in the right and left carotid plaques of 20 w HFD‐induced mice at 2 h post‐injection of pep‐CDs and maintained at high levels until the end of the 24‐h experimental period. Compared with 20 w HFD‐induced mice, due to the reduction of foam cells, PA signals from the right and left carotid plaques were relatively weaker in 10 w HFD‐induced mice. As depicted in Figure 6G,H, the bright FL signal was observed at the aortic and aortic arch plaque lesions from 20 w HFD‐induced mice at 2 h after injection of pep‐CDs and more intense than those from 10 w HFD‐induced mice. These results confirmed that pep‐CDs could serve as a multifunctional diagnostic agent to real‐time monitor AS pathological progression in vivo through PA/FL imaging.

Finally, to detailedly clarify the monitoring mechanism of pep‐CDs on AS progress, F4/80 immunofluorescence imaging of aortic root and FL imaging of isolated aorta were performed. As shown in Figure 6I, as the HFD feeding time increasing, the expression level of F4/80 gradually raised, indicating that the accumulation of macrophage foam cells in plaque increased with the delay of AS progression. Surprisingly, with the increase content of foam cells, the red fluorescence in the plaque region was significantly enhanced, and the degree of co‐localization of red fluorescence and F4/80 in the plaque region was improved, indicating the higher cumulant of pep‐CDs at the plaque lesion of 20 w HFD‐induced mice than that at the plaque lesion of 10 w HFD‐induced mice. In addition, it was also found that FL signal intensity of pep‐CDs at the aortic plaque lesion of 20 w HFD‐induced mice was extremely obvious and significantly higher than that of 10 w HFD‐induced mice (Figure 6J). This finding was mainly attributed to augmented plaques and foam cells from the aorta in the later AS pathological progression. Overall, pep‐CDs could be used to monitor AS progression through their amplified accumulation in the plaques of aorta.

### 2.8 In Vivo Treatment of AS with pep‐CDs

**Figure 7:**
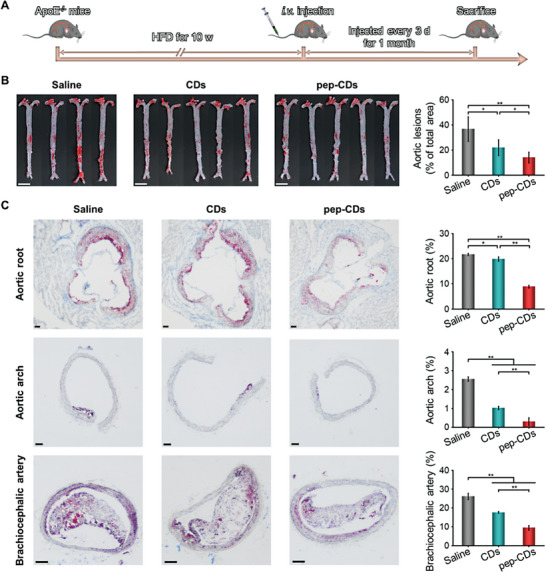
In vivo therapeutic effect of pep‐CDs nanozyme. A) Schematic diagram of the treatment protocol for AS. B) En face ORO staining images and corresponding quantitative analysis of the lesion area of aortas from ApoE^−/−^ mice after different treatments (scale bar: 5 mm). C) ORO staining images and corresponding quantitative analysis of the plaque area of aortic root, aortic arch, and brachiocephalic artery cryosections from ApoE^−/−^ mice after different treatments (scale bar: 100 µm). Data in (C) were illustrated as mean ± s.d. (*n* = 4–5). *p < 0.05, **p < 0.01.

Inspired by the favorable therapeutic performance in vitro of pep‐CDs, their anti‐AS treatment efficacy in vivo was further explored. ApoE^−/−^ mice were randomly divided into three groups after feeding HFD for 10 w. While maintaining HFD, the following treatments were given for 1 month (**Figure** **7**A): i) Saline group (i.v. injection of normal saline); ii) CDs group (i.v. injection of CDs (100 µL, 2 mg mL^‐1^)), and iii) pep‐CDs group (i.v. injection of pep‐CDs (100 µL, 2 mg mL^‐1^)). The therapeutic efficacy of each group was reported by ORO staining of the aorta and cryosections of the aortic root, aortic arch, and brachiocephalic artery. As revealed in Figure 7B, the large ORO‐positive regions were observed in the saline group, while a significant inhibition of plaque formation was exhibited in the CDs group, which could be attributed to the antioxidant, ROS scavenging, and SOD‐like activities of free CDs. Expectedly, pep‐CDs group revealed the fewest ORO‐stained areas and the most effective inhibition of plaque formation. The highest therapeutic efficacy should be arisen from PS‐mediated precision therapy. Quantitatively, there were significant differences between the various treatment groups. Consistent with the above results, the observation of ORO‐stained cryosections from the aortic root, aortic arch, and brachiocephalic artery also showed the most significant anti‐AS activity for pep‐CDs (Figure 7C). Together, these results indicated that pep‐CDs could fully exert the antioxidant, ROS scavenging, and SOD‐like activities of CDs through the target accumulation in AS lesion, thereby effectively reducing the AS development.

Furthermore, the composition of AS plaques in each treatment group was examined by histochemical analysis of the aortic root cryosections (Figure S19, Supporting Information). In hematoxylin and eosin (H&E) staining, the necrotic core area of pep‐CDs group was prominently smaller than that of saline and CDs groups. Masson's trichrome staining exhibited that the significantly higher concentration of collagen around the plaque in the pep‐CDs group, leading to the increased fiber cap thickness. Moreover, separate staining of anti‐F4/80 antibody and anti‐matrix metalloproteinase‐9 (MMP‐9) antibody showed that pep‐CDs could effectively reduce the number of foam cells and the expression of MMP‐9 in plaques. α‐smooth muscle actin (α‐SMA) antibody staining displayed the most serious VSMC accumulation from plaque in the pep‐CDs group. Since necrotic core area, foam cell infiltration level, and MMP‐9 expression were positively correlated with plaque development, while collagen concentration and VSMC accumulation were negatively related to AS severity, suggesting that pep‐CDs could significantly stabilize AS plaques. As a result, it was more convincing to prove that the target accumulation ability in AS conferred by PS‐specific peptide modification was conducive to energize pep‐CDs for effective inhibiting plaque formation.

### 2.9 In Vivo Therapeutic Mechanism for AS

**Figure 8:**
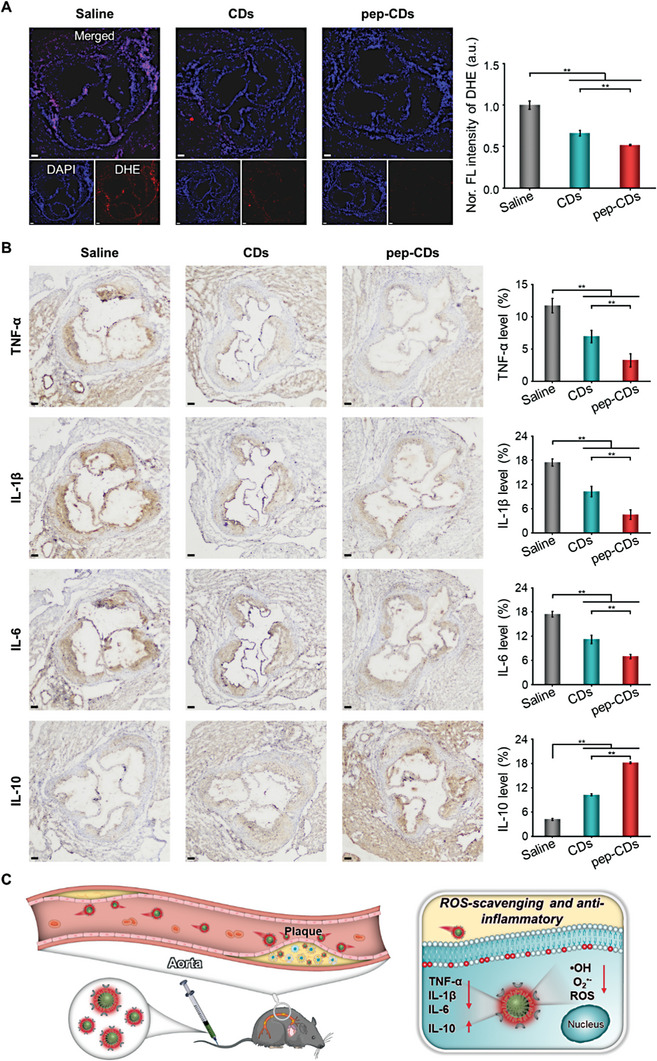
In vivo therapeutic mechanism of pep‐CDs nanozyme for AS. A) FL images and corresponding quantitative analysis of FL signal intensities of DHE‐stained aortic root cryosections from ApoE^−/−^ mice after different treatments. B) TNF‐α, IL‐1β, IL‐6, and IL‐10 immunohistochemistry staining images and corresponding quantitative analysis of the plaque TNF‐α, IL‐1β, IL‐6, and IL‐10 expression levels of aortic root cryosections from ApoE^−/−^ mice after different treatments. C) Schematic diagram of the therapeutic mechanism of pep‐CDs nanozyme for AS in vivo. All scale bars: 100 µm. Data in (A, B) were illustrated as mean ± s.d. (*n* = 4–5). **p < 0.01.

To further investigate the underlying anti‐AS mechanism of pep‐CDs in vivo, the ROS levels in plaques of each treatment group were assessed by dihydroethidium (DHE) staining from the aortic root cryosections. As depicted in **Figure** **8**A, the red fluorescence from the plaque lesion in the Saline group was the brightest, followed by that in the CDs group, and the weakest in the pep‐CDs group, indicating that pep‐CDs could effectively reduce the ROS level in plaque lesion, thus significantly alleviating oxidative stress. Since inflammatory factors play an important role in the development of AS, the levels of pro‐inflammatory and anti‐inflammatory factors in plaque tissue were measured. In fact, the expression of the pro‐inflammatory factors TNF‐α, IL‐1β, and IL‐6 at the plaque lesion was lowest in the pep‐CDs group, while comparable levels were monitored from the plaque area in the saline and CDs groups (Figure 8B). Contrary to this result, plaque lesion in the pep‐CDs group exhibited the least expression of the anti‐inflammatory factor IL‐10. Overall, pep‐CDs could effectively alleviate oxidative stress and inflammation by reducing ROS levels and the recruitment of pro‐inflammatory factors and enhancing the supplement of anti‐inflammatory factors in the microenvironment of AS plaques for anti‐AS applications (Figure 8C).

### 2.10 In Vivo Safety Evaluation

Considering the in vivo safety as the main basis for the biological application of nanomaterials, systemic toxicity of pep‐CDs was evaluated after 1 month of treatment. During the entire monitoring period, the weight of mice in each treatment group remained stable (Figure S20, Supporting Information). Clinical biochemical analysis exhibited that the levels of alanine aminotransferase (ALT), aspartate aminotransferase (AST), blood urea nitrogen (BUN), and serum creatinine (CREA) from mice in all groups were within the normal range (Figure S21, Supporting Information), proving that different nanotherapeutics had no significant side effect on liver and kidney function. In addition, the levels of white blood cells (WBC), red blood cells (RBC), hemoglobin (HGB), and platelets (PLT) in whole blood of various groups were detected by hematological parameter analysis. As presented in Figure S22 (Supporting Information), the number of WBC, RBC, and PLT in the Saline group exceeded the normal range except for HGB. Compared with saline group, WBC and RBC in CDs group returned to normal range. Surprisingly, the number of WBC, RBC, HGB, and PLT in the pep‐CDs group was within normal values. The contents of total cholesterol (TC) and low‐density lipoprotein (LDL) in CDs and pep‐CDs groups were lower than those in saline group, while triglyceride (TG) and high‐density lipoprotein (HDL) did not change significantly ( Figure S23, Supporting Information). H&E staining analysis demonstrated that no apparent injuries were found in the major organs of mice in each group (Figure S24, Supporting Information), indicating the low toxicity of nanotherapeutics in vivo. All these results proved that pep‐CDs could be safely applied for in vivo diagnosis and therapy due to their minimal toxic side effects and favorable biocompatibility.

## 3 Conclusion

In summary, a targeting theranostic nanoplatform (i.e., pep‐CDs) was constructed by simple coupling of PS‐specific peptide CLIKKPF and multifunctional CDs. The favorable PA/FL response, efficient antioxidant, ROS scavenging, SOD‐like and anti‐inflammatory activities of pep‐CDs inherited from CDs enabled precise dual‐mode PA and FL imaging‐guided AS therapy in vitro and in vivo. Noteworthily, the results of in vitro experiments confirmed that pep‐CDs were endowed with the specific recognition ability to foam cells via the introduced CLIKKPF target functional peptide. Considering that this specific recognition ability could positively enhance the accumulation of pep‐CDs at the plaque lesion, the theranostic efficacy of CDs was maximized. This work not only proposed a simple and feasible strategy for preparing the theranostic agents employing only a single functional unit (i.e., multifunctional CDs), but also importantly provided the active target adjuvant methods for effectively boosting safe and efficient theranostic.

## 4 Experimental Section

### Materials

Glutathione (GSH), *N*‐hydroxysuccinimide ester (BMPS), 1,1‐diphenyl‐2‐picrylhydrazyl radical (DPPH^•^), potassium persulfate (K_2_S_2_O_8_), ferrous sulfate heptahydrate (FeSO_4_•7H_2_O), β‐nicotinamide adenine dinucleotide reduced disodium salt (β‐NADH), nitrotetrazolium blue chloride (NBT), phenazine methosulfate (PMS), lipopolysaccharide (LPS), oil red O (ORO), isopropyl alcohol, calcium chloride (CaCl_2_), and 4‐(2‐hydroxyethyl)−1‐piperazineethanesulfonic acid (HEPES) were purchased from Aladdin Biochemical Technology Co., Ltd (Shanghai, China). Formamide was acquired from Sinopharm Chemical Reagent Co., Ltd. (Shanghai, China). Dimethyl sulfoxide (DMSO) was obtained from Chengdu Kelong Chemical Co., Ltd. (Chengdu, China). Phosphate buffered saline powder (PBS; 0.01 M, pH 7.2–7.4) was bought from SUMMUS Biological Technology Co., Ltd. (Harbin, China). Peptide CLIKKPF was purchased from Nanjing TGpeptide Biotechnology Co., Ltd. (Nanjing, China). Ethanol anhydrous, DMSO‐d6, and hydrogen peroxide solution (H_2_O_2_) were acquired from Shanghai Macklin Biochemical Co., Ltd. (Shanghai, China). 2,2′‐azinobis(3‐ethylbenzothiazoline‐6‐sulfonic acid ammonium salt) (ABTS) was obtained from Shanghai Yuanye Bio‐Technology Co., Ltd. (Shanghai, China). Salicylic acid (SA), sodium chloride (NaCl), 2,7‐dichlorofluorescin diacetate (DCFH‐DA), and hoechst 33258 were bought from Solarbio Science & Technology Co., Ltd. (Beijing, China). Fetal bovine serum (FBS), penicillin‐streptomycin, Dulbecco's modified Eagle's medium (DMEM), and RPMI 1640 medium were purchased from Thermo Fisher Biochemical Product (Beijing) Co., Ltd. (Beijing, China). Oxidized‐low‐density lipoprotein (ox‐LDL) was bought from Guangzhou Yiyuan Biotech. Co. Ltd. (Guangzhou, China). 4% Paraformaldehyde was acquired from Beijing Labgic Technology Co., Ltd. (Beijing, China). Cell Proliferation Assay Kit (MTS) was obtained from Saint‐Bio Biotechnology Co., Ltd. (Shanghai, China).

### Characterizations

The morphologies of the samples were observed by a Talos F200S TEM (ThermoFisher, Czech Rep). The hydrodynamic diameters and zeta potentials were measured on a Zetasizer Nano ZS 90 unit (Malvern, UK). X‐ray photoelectron spectroscopy (XPS) was performed on a K‐Alpha photoelectron spectrometer (ThermoScientific, UK). FT‐IR spectra were obtained from a Nicolet iS50 FT‐IR spectrometer (ThermoFisher, UK). ^1^H NMR spectra were recorded on an AVANCE NEO 400 spectrometer (Bruker, Switzerland, DMSO‐*d6* as solvent). UV–Vis absorption spectra were measured using a UV‐6100 ultraviolet spectrometer (Metash, China). FL spectra were acquired from a RF‐6000 fluorospectrophotometer (Shimadzu, Japan). The purity of the material was determined by a HPLC‐MS/MS method (Agilent, UK). In vitro and in vivo PA images and data were collected by a VEVO LAZR‐X small animal photoacoustic imaging system (Fujifilm VisualSonics, UK). In vitro and in vivo FL images and data were obtained by an IVIS Lumina III small animal optical imaging system (PerkinElmer, UK). MTS results were acquired from a microplate reader (BioTek, UK). Cell uptake was observed by a CytoFLEX LX flow cytometry (Beckman Coulter, UK). Cell FL images were captured on a TCS SP8 DIVE CLSM (Leica, Germany). Cell staining images were obtained from a IX71 inverted fluorescence microscope (Olympus, Japan).

### Synthesis: Synthesis of CDs

CDs were prepared through a simple one‐pot solvothermal method. Briefly, 0.5 g of GSH was fully dissolved in 15 mL of formamide. The transparent mixture was then transferred into a Teflon autoclave and heated in an oven at 160 °C for 4 h. After the autoclave temperature was cooled to room temperature naturally, the obtained dark green solution was diluted with 30 mL of deionized water. Thereafter, the diluted solution was filtered with a 0.22 µm syringe filter to remove larger particles and dialyzed (molecular weight cut‐off (MWCO) 3500 Da) with deionized water for 1 w to get rid of unreacted materials and small molecular weight reaction products. Finally, the purified CDs were gathered as dark green powder through lyophilization.

### Synthesis of pep‐CDs

BMPS were employed as a heterobifunctional crosslinking reagent to functionalize CDs with peptide CLIKKPF. In brief, CDs (40 mg) and BMPS (20 mg) were fully dissolved in 20 mL of mixture solution of PBS (0.01 m, pH 7.2‐7.4) and DMSO (V:V = 7:3) and kept stirring in an oil bath at 40 °C for 6 h. Then, purified maleimide‐modified CDs were obtained through dialysis (molecular weight cut‐off (MWCO) 3500 Da). Afterward, maleimide‐modified CDs (0.5 mg mL^‐1^ CDs) and peptide CLIKKPF (0.5 mg mL^‐1^) were subsequently dispersed in 32 mL of PBS (0.01 m, pH 7.2‐7.4) and kept stirring in an oil bath at 40 °C for 24 h. The obtained solution was dialyzed (molecular weight cut‐off (MWCO) 3500 Da) with deionized water for 2 d. Finally, the purified pep‐CDs were collected as dark green powder through lyophilization.

### Purity Analysis

The purity of the material was determined by an HPLC‐MS/MS method. Chromatographic separation was achieved by elution conditions (eluent A: water with 0.1% formic acid, eluent B: acetonitrile) of mobile phase A:mobile phase B = 50:50. The column temperature was maintained at 35 °C. The injection volume was 20 µL and the flow rate was 0.3 mL min^−1^. All targets were resolved and identified based on the precursor ion, product ion, and retention time in a 1 min run time. First, appropriate monitoring ions and corresponding detection parameters were selected. Next, the prepared peptide standard solution was measured in order of concentration from low to high. Subsequently, the standard curve regression equation was obtained with the chromatographic peak area as the vertical coordinate and the concentration as the horizontal coordinate. Finally, the content of free peptides in pep‐CDs samples was determined three times by the established method and calculated by the standard curve regression equation.

### Antioxidant Activity Assay: DPPH^•^‐Scavenging Activity Assay

The DPPH^•^ scavenging capability was measured using the previously established protocol with some modifications.^[^ ^31^ ^]^ Briefly, DPPH^•^ ethanol solution (0.3 mg mL^‐1^) was blended with different concentrations of samples and incubated at 37 °C in the dark for 30 min. Subsequently, the absorbance of all samples at 540 nm was recorded, and the DPPH^•^ elimination efficiency was reckoned.

### ABTS^•+^‐Scavenging Activity Assay

The ABTS^•+^ scavenging capability was determined according to the previous report with minor modifications.^[^ ^32^ ^]^ First, the ABTS^•+^ solution was produced by adding ABTS solution (6.8 × 10^‐3^ m) to an equal volume of K_2_S_2_O_8_ (2.4 × 10^‐3^ m) and incubating at room temperature in the dark for 12 h. Then, the ABTS^•+^ working solution was prepared by diluting the above ABTS^•+^ solution to twofold with deionized water. Afterward, the ABTS^•+^ working solution was mixed with different concentrations of samples and incubated at 37 °C in the dark for 10 min. Finally, the absorbance of all samples at 734 nm was measured, and the ABTS^•+^ elimination efficiency was calculated.

### ^•^OH‐Scavenging Activity Assay

The ^•^OH scavenging capability was evaluated using the reported method with some modifications.^[^ ^33^ ^] •^OH was generated through the classical Fenton reaction. Firstly, FeSO_4_ solution (18 × 10^‐3^ m), SA ethanol solution (18 × 10^‐3^ m), and different concentrations of samples were prepared and mixed. Then, H_2_O_2_ solution (176 × 10^‐3^ m) was added to the above mixture solution and incubated at 37 °C in the dark for 20 min. Finally, the absorbance of all samples at 520 nm was recorded, and the ^•^OH elimination efficiency was reckoned.

### SOD‐Like Activity Assay

The SOD‐like activity was studied by the previously reported photochemical reduction of NBT in the NADH‐NBT‐PMS system with minor modifications.^[^ ^34^, ^35^ ^]^ Briefly, the different concentrations of samples were added to 2.8 mL Tris‐HCl buffer (16 × 10^‐3^ m, pH 8.8) containing 1.81 × 10^‐3^ m β‐NADH and 0.38 × 10^‐3^ m NBT. Subsequently, the reaction was triggered by the addition of 0.2 mL of PMS (2.25 × 10^‐3^ m in 16 × 10^‐3^ m Tris‐HCl buffer, pH 8.0) and incubated at room temperature for 5 min. Finally, the absorbance of all samples at 560 nm was measured, and the O_2_^•−^ elimination efficiency was calculated.

### PA/FL Dual‐Modality Imaging Performance of pep‐CDs: PA Imaging Property of pep‐CDs

1 mg mL^‐1^ pep‐CDs solution was prepared and placed into a tetrafluoro capillary tube with an inner diameter of 0.3 mm and an outer diameter of 0.6 mm. Then, the tube was immersed in water medium and irradiated with wavelengths ranging from 680 to 970 nm. Subsequently, the PA signal was collected via the VEVO LAZR‐X photoacoustic imaging system. In addition, the PA images and signals at excitation wavelength of 685 nm of pep‐CDs solutions with different concentrations (0, 0.1, 0.2, 0.4, 0.6, 0.8, and 1 mg mL^‐1^) were collected with the above same operation.

### FL Imaging Property of pep‐CDs

The pep‐CDs solutions with different concentrations (0, 1, 2, 4, 6, 8, and 10 µg mL^‐1^) were prepared and dropped into a 96‐well plate. Subsequently, the FL images and signals were collected via the IVIS optical imaging system.

### Cell Culture

Three kinds of cell lines were involved in this study. RAW264.7 and MOVAS were cultured in DMEM and added with 10% FBS and 1% penicillin‐streptomycin. HUVEC was cultured in RPMI‐1640 medium supplemented with 10% FBS and 1% penicillin‐streptomycin. All cells were cultured in a humidified incubator at 37 °C in 5% CO_2_ atmosphere.

### In Vitro Cytotoxicity Evaluation

The in vitro cytotoxicity was characterized via MTS assay. Typically, RAW264.7, HUVEC, and MOVAS cells were seeded in 96‐well plates and allowed to adhere all night. Afterward, the culture medium was replaced with fresh medium containing CDs or pep‐CDs ranging from 0 to 400 µg mL^‐1^. After 12 or 24 h of incubation, 10 µL MTS solution was added into each well of plate and cocultured with the cells for 2 h. Finally, the absorbance at 490 nm was measured to calculate the in vitro cytotoxicity.

### In Vitro Formation of Foam Cells

ORO staining was used here to characterize the formation of foam cells via coloring the lipid. In brief, RAW264.7 cells were seeded in a 24‐well plate and allowed to adhere all night. Cells were first incubated with LPS (1 µg mL^‐1^) for 12 h and treated with ox‐LDL (80 µg mL^‐1^) for another 12, 24, or 48 h to form macrophage‐derived foam cells. After washing with PBS for three times and fixing with 4% paraformaldehyde for 30 min, the cells were stained with 0.3% ORO working solution for 20 min. Finally, the cells were washed again with 60% isopropyl alcohol for three times and observed by an IX71 inverted fluorescence microscope.

### In Vitro PS‐Mediated Specific Targeting Recognition Ability toward Foam Cells

PS‐mediated specific targeting recognition ability on foam cells was investigated through cell imaging experiments. Briefly, RAW264.7 cells were seeded in glass confocal culture dishes and waited to adhere overnight. In ox‐LDL (+) group, cells were incubated with LPS (1 µg mL^‐1^) for 12 h and treated with ox‐LDL (80 µg mL^‐1^) for another 48 h, while in ox‐LDL (‐) group cells were incubated without LPS and ox‐LDL. Then, cells were incubated with PBS, CDs (100 µg mL^‐1^), or pep‐CDs (100 µg mL^‐1^) for 4 h. For blocked group, cells were cultured with excess peptide CLIKKPF (100 µg mL^‐1^) for 2 h prior to incubation with pep‐CDs (100 µg mL^‐1^) for 4 h. Subsequently, the cells were washed with Ca^2+^ binding buffer (10 × 10^‐3^ m HEPES, pH 7.4; 140 × 10^‐3^ m NaCl; 2.5 × 10^‐3^ m CaCl_2_) for three times and fixed by 4% paraformaldehyde (containing an additional 2.5 × 10^‐3^ m of CaCl_2_) for 30 min. Afterward, the cells were incubated with 5 µg mL^‐1^ Hoechst 33258 for 5 min for nuclei staining. Finally, the cells were observed using a CLSM.

For the foam cell imaging at different time points, sample preparation procedure was similar as which mentioned above except that after induction with LPS and ox‐LDL cells were incubated with pep‐CDs (100 µg mL^‐1^) for 0.5, 1, or 4 h.

### In Vitro Imaging of Cells with Different Degrees of Foaming

RAW264.7 cells were seeded in glass confocal culture dishes and allowed to adhere overnight. Cells were first incubated with LPS (1 µg mL^‐1^) for 12 h and treated with ox‐LDL (80 µg mL^‐1^) for another 0, 12, 24, or 48 h. Then, cells were incubated with pep‐CDs (100 µg mL^‐1^) for 4 h. Subsequently, the cells were washed with Ca^2+^ binding buffer for three times and fixed by 4% paraformaldehyde (containing an additional 2.5 × 10^‐3^ m of CaCl_2_) for 30 min. Afterward, the cells were incubated with 5 µg mL^‐1^ Hoechst 33258 for 5 min for nuclei staining. Finally, the cells were observed using a CLSM.

### Cellular Uptake

RAW264.7 cells were seeded in a six‐well plate and allowed to adhere overnight. Cells were first incubated with PBS, CDs (100 µg mL^‐1^), and pep‐CDs (100 µg mL^‐1^) for 4 h. Subsequently, cells in each well were collected. Finally, the cellular uptake of CDs and pep‐CDs was quantified by a flow cytometry.

### Intracellular ROS Scavenging in RAW264.7 Cells

The intracellular accumulation of ROS was monitored by the fluorescent probe DCFH‐DA. In brief, RAW264.7 cells were seeded in glass confocal culture dishes and allowed to adhere overnight. Then, the cells were treated with PBS, CDs (50 and 100 µg mL^‐1^), or pep‐CDs (50 and 100 µg mL^‐1^) for 4 h, respectively. Subsequently, the cells were stimulated by 1 µg mL^‐1^ LPS for 1.5 h. Afterward, the cells were incubated with 10 × 10^‐6^ m DCFH‐DA for 30 min and fixed by 4% paraformaldehyde for another 30 min. Finally, the cells were observed using a CLSM.

### In Vitro Resistance on Foam Cell Formation

RAW264.7 cells were seeded in 24‐well plate and allowed to adhere all night. Cells were first incubated with LPS (1 µg mL^‐1^) for 12 h. Then, the cells were treated with PBS, CDs (50 and 100 µg mL^‐1^), or pep‐CDs (50 and 100 µg mL^‐1^) for 6 h. Subsequently, the cells were stimulated by 80 µg mL^‐1^ ox‐LDL for 24 h. The normal control group was treated with fresh medium. After washing with PBS for three times and fixing with 4% paraformaldehyde for 30 min, the cells were stained with 0.3% ORO working solution for 20 min. Finally, the cells were washed again with 60% isopropyl alcohol for three times and observed by an IX71 inverted fluorescence microscope.

### Establishment of AS Model Mice

The ApoE^−/−^ mice with 6 week old and and male C57BL/6 mice (6–8 weeks) were obtained from Jiangsu Huachuang Sino Pharmatech Co., Ltd. (Taizhou, China). All the animal experiments were performed according to the regulations approved by the Institutional Animal Care and Use Committee of Chongqing University (Issue No.: CQULA‐2022JC‐12‐016). The ApoE^−/−^ mice were fed with HFD for either 10 or 20 w to induce AS models with different pathological progression.

### Hemolysis Study

Blood compatibility of pep‐CDs was evaluated through hemolysis test. Briefly, the red blood cells were gathered from whole blood of healthy C57BL/6 mice. After being washing with PBS for three times by centrifugation (10000 rpm, 5 min), red blood cells were divided evenly into eight groups and experienced different treatments: i) H_2_O (positive control group), ii) PBS (negative control group), and iii–viii) different concentrations of pep‐CDs (6.25, 12.5, 25, 50, 100, and 200 µg mL^‐1^), respectively. After incubation at 37 °C for 6 h, the supernatant of all samples was collected by centrifugation. Subsequently, The absorbance of the supernatant was measured at 540 nm. Finally, hemolysis rates were calculated by the following equation:

(1) $$\mathrm{Hemolysis}\;\mathrm{rate}\left(\%\right)=\frac{A_{t}-A_{\mathrm{nc}}}{A_{\mathrm{pc}}-A_{\mathrm{nc}}}\times 100\%$$

where *A*_t_, *A*_nc_, and *A*_pc_ represent the absorbance of experimental group, negative control group, and the positive control group at 540 nm, respectively.

### In Vivo Plaque Targeting Ability

The plaque targeting ability in vivo was studied via PA/FL dual‐modality imaging. In brief, the ApoE^−/−^ mice were fed with HFD for 10 w. Then, mice were separated into three groups, CDs (100 µL, 2 mg mL^‐1^), pep‐CDs (100 µL, 2 mg mL^‐1^), and pep+pep‐CDs (100 µL, 2 mg mL^‐1^) were intravenously injected into mice of each group, respectively. Particularly, in the pep+pep‐CDs group, mice were intravenously injected with excessive peptide CLIKKPF (100 µL, 2 mg mL^‐1^) 2 h before being intravenously injected with pep‐CDs. Finally, in vivo PA imaging at an excitation wavelength of 685 nm and FL imaging were performed on the IVIS optical imaging system and VEVO LAZR‐X photoacoustic imaging system at 0, 2, 4, 8, 12, and 24 h postinjection.

For ApoE^−/−^ mice fed with HFD for 20 w, the treatment procedure after injection of pep‐CDs (100 µL, 2 mg mL^‐1^) was similar to the above.

### In Vivo Pharmacokinetics Study

C57BL/6 mice were intravenously administrated with pep‐CDs (100 µL, 2 mg mL^‐1^). Then, whole blood samples of equal volume were collected at different time points (0, 0.5, 1, 2, 4, 8, 12, and 24 h). Finally, the fluorescence signals of blood samples were determined by the IVIS optical imaging system and the pharmacokinetics was calculated.

### In Vivo Tissue Distributions

In vivo tissue distribution of pep‐CDs was examined after intravenous injection in ApoE^−/−^ mice (after 10 w of HFD). First, mice were intravenously administrated with pep‐CDs (100 µL, 2 mg mL^‐1^). Then, heart, liver, spleen, lung, and kidney tissues were collected at different time points (0, 0.5, 1, 2, 4, 8, 12, and 24 h). Finally, the fluorescence signals of tissues were recorded by the IVIS optical imaging system.

### Ex Vivo FL Imaging

The plaque recognition ability was examined via FL imaging of isolated aorta. Briefly, the ApoE^−/−^ mice were fed with HFD for 10 w. Then, mice were separated into three groups, CDs (100 µL, 2 mg mL^‐1^), pep‐CDs (100 µL, 2 mg mL^‐1^), and pep (100 µL, 2 mg mL^‐1^) + pep‐CDs (100 µL, 2 mg mL^‐1^) were intravenously injected into mice of each group, respectively. After 2 h post‐injection, mice were euthanized and the aortas were removed. Finally, the FL signals of aortic tissues were collected via the IVIS optical imaging.

For C57BL/6 mice, the treatment procedure after injection of CDs (100 µL, 2 mg mL^‐1^) was similar to the above.

For ApoE^−/−^ mice fed with HFD for 20 w, the treatment procedure after injection of pep‐CDs (100 µL, 2 mg mL^‐1^) was similar to the above.

### In Vivo Anti‐AS Effect

For estimation of in vivo anti‐AS effect, 15 ApoE^−/−^ mice fed with HFD for 10 w were randomly divided into three groups and received the following treatments for one month: i) intravenously injected with normal saline, ii) intravenously injected with CDs (100 µL, 2 mg mL^‐1^), iii) intravenously injected with pep‐CDs (100 µL, 2 mg mL^‐1^). All formulations were intravenously injected once every 3 d. Mouse body weight was recorded regularly during the treatment.

### In Vivo Biosafety Evaluation

The biocompatibility in vivo was investigated via blood routine test, serum biochemistry analysis, and H&E staining. After different treatments, the blood and major organs ((heart, liver, spleen, lung, and kidney) of mice were collected. The levels of RBC, WBC, PLT, and HGB in complete blood were analyzed via blood routine test. The levels of ALT, AST, BUN, CREA, TC, LDL, TG, and HDL in the separated serum were also measured via biochemistry analysis. Finally, the major organs were embedded, sliced, and stained by H&E for histological analysis.

### ORO Staining

Aortic AS plaque areas were assessed by ORO staining. After collecting the aorta, the aorta was longitudinally incised and stained with ORO. Additionally, cryosections of the aortic root, aortic arch, and brachiocephalic artery were prepared and then stained with ORO.

### Histology and Immunohistochemistry

After collecting the aortic roots, cryosections of the aortic roots were prepared, and then stained with H&E and Masson's trichrome. For immunohistochemistry analysis, cryosections were incubated with antibodies, including F4/80, MMP‐9, α‐SMA, TNF‐α, IL‐1β, IL‐6, and IL‐10.

### Histology and Immunohistochemistry

Data were revealed as mean ± standard deviation (s.d.). Student's *t*‐test was employed for the comparative analysis of the statistical significance among different groups (*p < 0.05 represents for significant difference and **p < 0.01 for very significant difference).

## Conflict of Interest

The authors declare no conflict of interest.

## Supporting information

Supporting Information

## Data Availability

The data that support the findings of this study are available on request from the corresponding author. The data are not publicly available due to privacy or ethical restrictions.

## References

[1] J. Frostegård , BMC Med. 2013, 11, 117. 23635324 10.1186/1741-7015-11-117 PMC3658954

[2] Y. Wang , L. Li , W. Zhao , Y. Dou , H. An , H. Tao , X. Xu , Y. Jia , S. Lu , J. Zhang , H. Hu , ACS Nano 2018, 12, 8943. 30114351 10.1021/acsnano.8b02037

[3] X. Ge , H. Cui , J. Kong , S.‐Y. Lu , R. Zhan , J. Gao , Y. Xu , S. Lin , K. Meng , L. Zu , S. Guo , L. Zheng , Adv. Mater. 2020, 32, 2000037. 10.1002/adma.202000037 32803803

[4] A. J. Lusis , Nature 2000, 407, 233. 11001066 10.1038/35025203 PMC2826222

[5] C. Weber , H. Noels , Nat. Med. 2011, 17, 1410. 22064431 10.1038/nm.2538

[6] B. X. Ma , Y. Xiao , Q. B. Lv , G. C. Li , Y. B. Wang , G. S. Fu , Adv. Mater. 2023, 35, 2206129. 10.1002/adma.202206129 36394179

[7] B. Ma , H. Xu , W. Zhuang , Y. Wang , G. Li , Y. Wang , Small 2020, 16, 2003253. 10.1002/smll.202003253 33078569

[8] Q. Chen , S. Sun , H. Lin , Z. Li , A. Wu , X. Liu , F.‐G. Wu , W. Zhang , ACS Appl. Bio Mater. 2021, 4, 2759. 10.1021/acsabm.0c01663 35014315

[9] S. Sun , Q. Chen , Y. Li , Y. Yu , Z. Li , H. Lin , SmartMat 2022, 3, 311.

[10] S. Sun , Q. Chen , Z. Tang , C. Liu , Z. Li , A. Wu , H. Lin , Angew. Chem., Int. Ed. 2020, 59, 21041. 10.1002/anie.202007786 32914924

[11] A. Lv , Q. Chen , C. Zhao , S. Li , S. Sun , J. Dong , Z. Li , H. Lin , Chin. Chem. Lett. 2021, 32, 3653.

[12] C. Liu , W. Fan , W.‐X. Cheng , Y. Gu , Y. Chen , W. Zhou , X.‐F. Yu , M. Chen , M. Zhu , K. Fan , Q.‐Y. Luo , Adv. Funct. Mater. 2023, 33, 2213856.

[13] Y. Zhang , W. Gao , Y. Ma , L. Cheng , L. Zhang , Q. Liu , J. Chen , Y. Zhao , K. Tu , M. Zhang , C. Liu , Nano Today 2023, 49, 101768.

[14] D. He , M. Yan , P. Sun , Y. Sun , L. Qu , Z. Li , Chin. Chem. Lett. 2021, 32, 2994.

[15] X. Sun , W. Li , X. Zhang , M. Qi , Z. Zhang , X.‐E. Zhang , Z. Cui , Nano Lett. 2016, 16, 6164. 27622963 10.1021/acs.nanolett.6b02386

[16] M. Naghavi , R. John , S. Naguib , M. S. Siadaty , R. Grasu , K. C. Kurian , W. B. Van Winkle , B. Soller , S. Litovsky , M. Madjid , J. T. Willerson , W. Casscells , Atherosclerosis 2002, 164, 27. 12119190 10.1016/s0021-9150(02)00018-7

[17] S. Zhang , Y. Liu , Y. Cao , S. Zhang , J. Sun , Y. Wang , S. Song , H. Zhang , Adv. Mater. 2022, 34, 2110660. 10.1002/adma.202110660 35238081

[18] M. F. Attia , N. Anton , J. Wallyn , Z. Omran , T. F. Vandamme , J. Pharm. Pharmacol. 2019, 71, 1185. 31049986 10.1111/jphp.13098

[19] H. Ikeda , A. Ishii , K. Sano , H. Chihara , D. Arai , Y. Abekura , H. Nishi , M. Ono , H. Saji , S. Miyamoto , Atherosclerosis 2018, 275, 1. 29852399 10.1016/j.atherosclerosis.2018.05.028

[20] R. Ji , X. Li , C. Zhou , Q. Tian , C. Li , S. Xia , R. Wang , Y. Feng , W. Zhan , Nanoscale 2018, 10, 20246. 30361722 10.1039/c8nr04703k

[21] M. Wu , X. Li , Q. Guo , J. Li , G. Xu , G. Li , J. Wang , X. Zhang , Nanomed.: Nanotechnol. Biol. Med. 2021, 32, 102330. 10.1016/j.nano.2020.102330 33171287

[22] T. Q. Nhan , W. C. Liles , A. Chait , J. T. Fallon , S. M. Schwartz , Arterioscler., Thromb., Vasc. Biol. 2003, 23, 1276. 12763761 10.1161/01.ATV.0000078602.54433.07

[23] B. R. Smith , J. Heverhagen , M. Knopp , P. Schmalbrock , J. Shapiro , M. Shiomi , N. I. Moldovan , M. Ferrari , S. C. Lee , Biomed. Microdevices 2007, 9, 719. 17562181 10.1007/s10544-007-9081-3

[24] K. G. Yang , M. Q. Jia , S. Cheddah , Z. Y. Zhang , W. W. Wang , X. Y. Li , Y. Wang , C. Yan , Bioact. Mater. 2022, 15, 343. 35356814 10.1016/j.bioactmat.2021.12.017 PMC8935132

[25] L. Pan , S. Sun , L. Zhang , K. Jiang , H. Lin , Nanoscale 2016, 8, 17350. 27714173 10.1039/c6nr05878g

[26] C. Zhao , S. Sun , S. Li , A. '. Lv , Q. Chen , K. Jiang , Z. Jiang , Z. Li , A. Wu , H. Lin , ACS Appl. Mater. Interfaces 2022, 14, 10142. 35175020 10.1021/acsami.2c00174

[27] M. Giorgio , M. Trinei , E. Migliaccio , P. G. Pelicci , Nat. Rev. Mol. Cell Biol. 2007, 8, 722. 17700625 10.1038/nrm2240

[28] M. N. Sack , F. Y. Fyhrquist , O. J. Saijonmaa , V. Fuster , J. C. Kovacic , J. Am. Coll. Cardiol. 2017, 70, 196. 28683968 10.1016/j.jacc.2017.05.034 PMC5551687

[29] L. Nagy , P. Tontonoz , J. G. A. Alvarez , H. Chen , R. M. Evans , Cell 1998, 93, 229. 9568715 10.1016/s0092-8674(00)81574-3

[30] X. Zang , M. Cheng , X. Zhang , X. Chen , J. Mater. Chem. B 2021, 9, 3284. 33881414 10.1039/d0tb02956d

[31] H. Wang , M. Zhang , Y. Ma , B. Wang , H. Huang , Y. Liu , M. Shao , Z. Kang , ACS Appl. Mater. Interfaces 2020, 12, 41088. 32805964 10.1021/acsami.0c11735

[32] L. Wang , M. Ma , Z. Yu , S.‐K. Du , Food Chem. 2021, 352, 129399. 33662918 10.1016/j.foodchem.2021.129399

[33] C. Zhou , J. Hu , H. Ma , A. E. A. Yagoub , X. Yu , J. Owusu , H. Ma , X. Qin , Food Chem. 2015, 187, 270. 25977026 10.1016/j.foodchem.2015.04.092

[34] J. V. Mehta , S. B. Gajera , M. N. Patel , Spectrochim. Acta Part A 2015, 136, 1881. 10.1016/j.saa.2014.10.103 25467683

[35] N. N. Sarvestani , F. Khodagholi , N. Ansari , M. M. Farimani , Phytomedicine 2013, 20, 939. 23639191 10.1016/j.phymed.2013.03.013

