# Supplementary material for: Target Functionalized Carbon Dot Nanozymes with Dual‐Model Photoacoustic and Fluorescence Imaging for Visual Therapy in Atherosclerosis
Source: Adv Sci (Weinh). 2023 Dec 25;11(6):2307441. doi: 10.1002/advs.202307441 (PMC10853701; doi:10.1002/advs.202307441)
Supplement: Supplementary file 1 — Supporting Information [file ADVS-11-2307441-s001.pdf]

ADVANCED SCIENCE
Open Access

Supporting Information

for *Adv. Sci.*, DOI 10.1002/advs.202307441

Target Functionalized Carbon Dot Nanozymes with Dual-Model Photoacoustic and Fluorescence Imaging for Visual Therapy in Atherosclerosis

Qiao Chen, Xinmei Duan, Yao Yu, Rongrong Ni, Guojing Song, Xu Yang, Li Zhu, Yuan Zhong, Kun Zhang, Kai Qu*, Xian Qin* and Wei Wu**

Supporting Information

Target functionalized carbon dot nanozymes with dual-model photoacoustic and fluorescence imaging for visual therapy in atherosclerosis

Qiao Chen[1], Xinmei Duan[1], Yao Yu[2], Rongrong Ni[3], Guojing Song[4], Xu Yang[1], Li Zhu[1], Yuan Zhong[1], Kun Zhang[1],[5],*, Kai Qu[1],[5],*, Xian Qin[1],[5],*, and Wei Wu[1],*

[1] Q. Chen, X. M. Duan, X. Yang, L. Zhu, Y. Zhong, K. Zhang, K. Qu, X. Qin, W. Wu

Key Laboratory for Biorheological Science and Technology of Ministry of Education, State and Local Joint Engineering Laboratory for Vascular Implants, Bioengineering College of Chongqing University, Chongqing, 400044, China.

E-mail: kunzh01@cqu.edu.cn (K. Zhang); qukaigood@cqu.edu.cn (K. Qu); qinxian224@cqu.edu.cn (X. Qin); david2015@cqu.edu.cn (W. Wu)

[2] Y. Yu

Thyroid Breast Surgery Department, Dazhou Central Hospital, Dazhou 635000, China.

[3] R. R. Ni

Medical Department, Southwest Hospital, Third Military Medical University, Chongqing, 400038, China.

[4] G. J. Song

Urology, Southwest Hospital, Third Military Medical University, Chongqing, 400038, China.

[5] K. Zhang, K. Qu, X. Qin

Chongqing University Three Gorges Hospital, Chongqing, 404000, China.

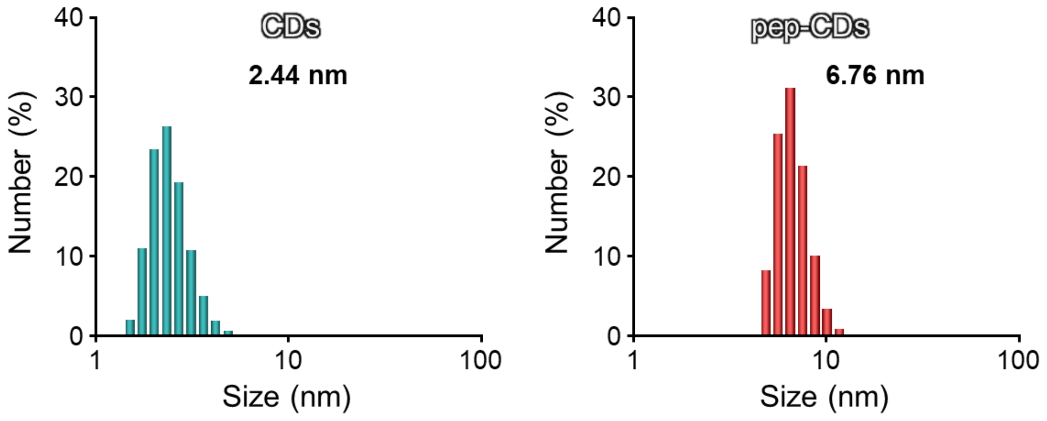

Figure S1. Hydrodynamic diameter of CDs and pep-CDs.

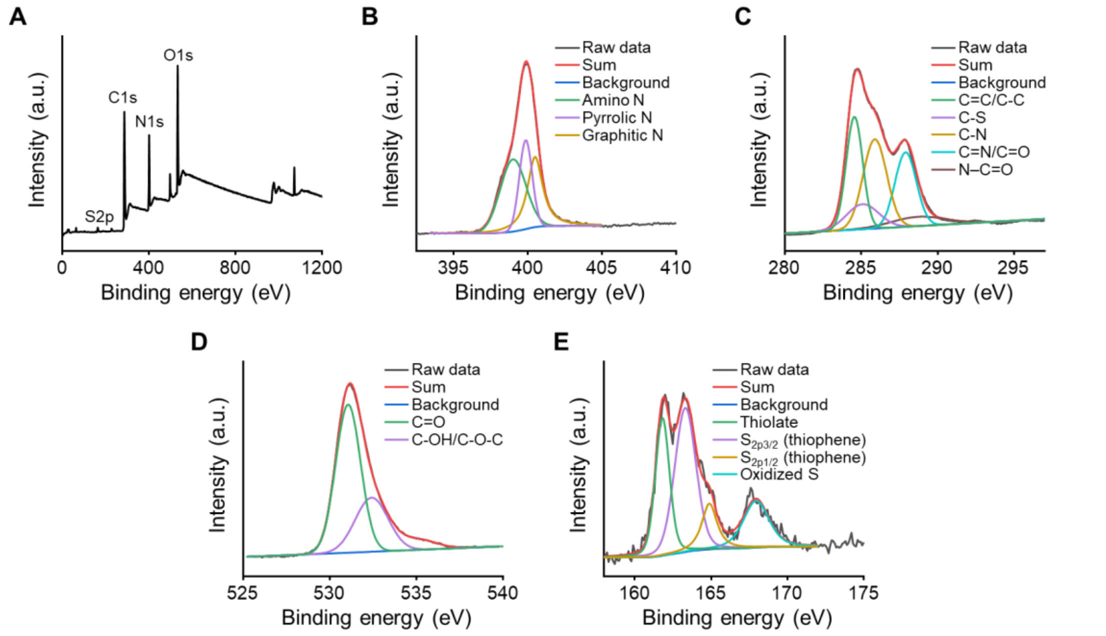

Figure S2. A) XPS survey spectra and high resolution B) N 1s, C) C 1s, D) O 1s, and E) S 2p spectra of CDs.

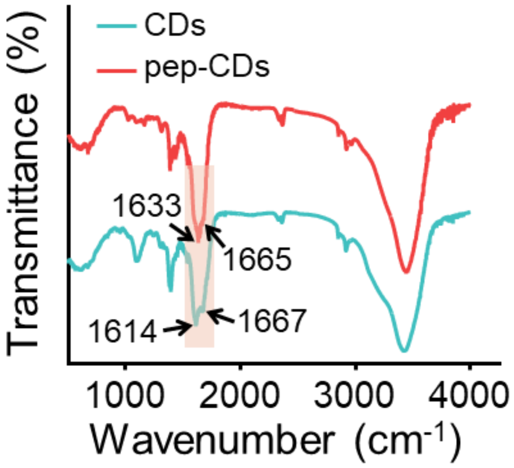

Figure S3. FT-IR spectra of CDs and pep-CDs.

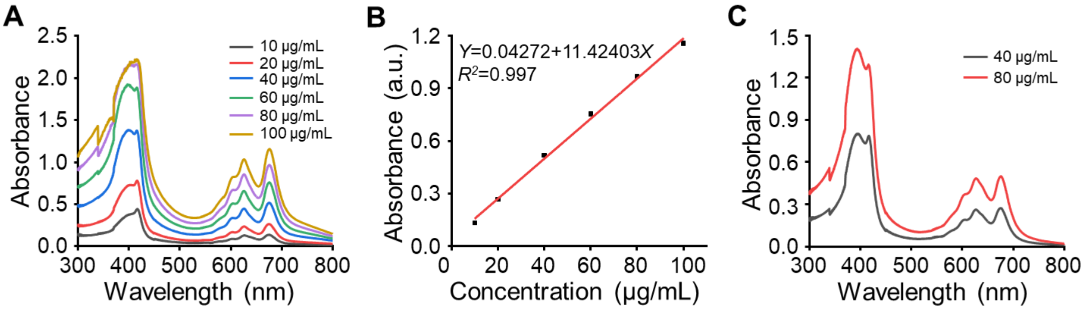

Figure S4. A) UV-Vis absorption spectra of CDs at different concentrations. B) Calibration curve of the absorption intensity of CDs at 675 nm versus the corresponding concentration. C) UV-Vis absorption spectra of pep-CDs at different concentrations.

Table S1. Monitoring ions and the corresponding detection parameters.

Compound	Quantitative ion pair (m/z)	Qualitative ion pair (m/z)	Collision energy (V)
Peptide	848.0755/586.3724	848.0755/391.2319<br>848.0755/257.1957<br>848.0755/242.1847	30

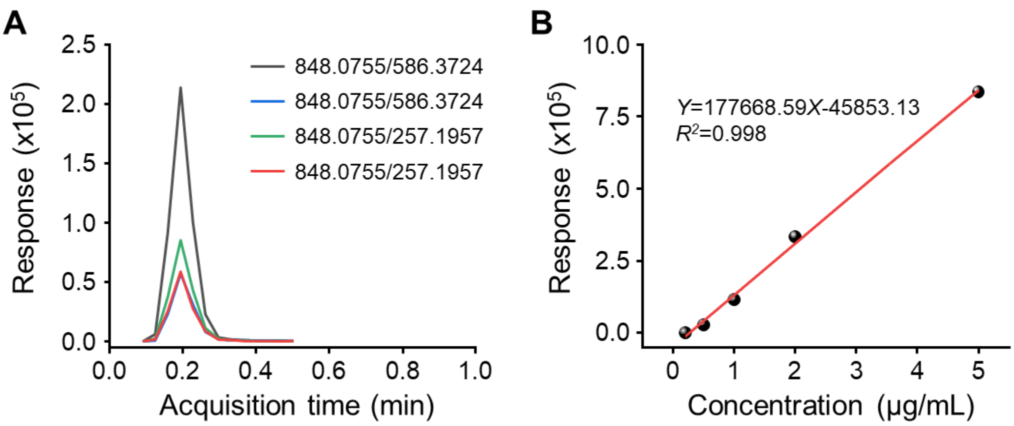

Figure S5. A) Qualitative and quantitative ion pair chromatogram of 5 μg/mL peptide standard solution. B) Calibration curve of the chromatographic peak area corresponding concentration.

Table S2. Detection result of the free peptides in pep-CDs.

Samples	Sample concentration (μg/mL)	Peptide detection concentration (μg/mL)	Content of free peptides in samples (%)
pep-CDs-1	10	0.2583	2.583
pep-CDs-2	10	0.2595	2.595
pep-CDs-3	10	0.2598	2.598

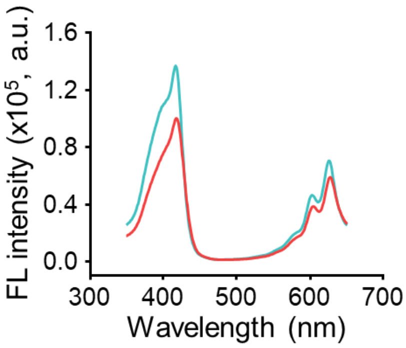

Figure S6. FL excitation spectra of CDs and pep-CDs.

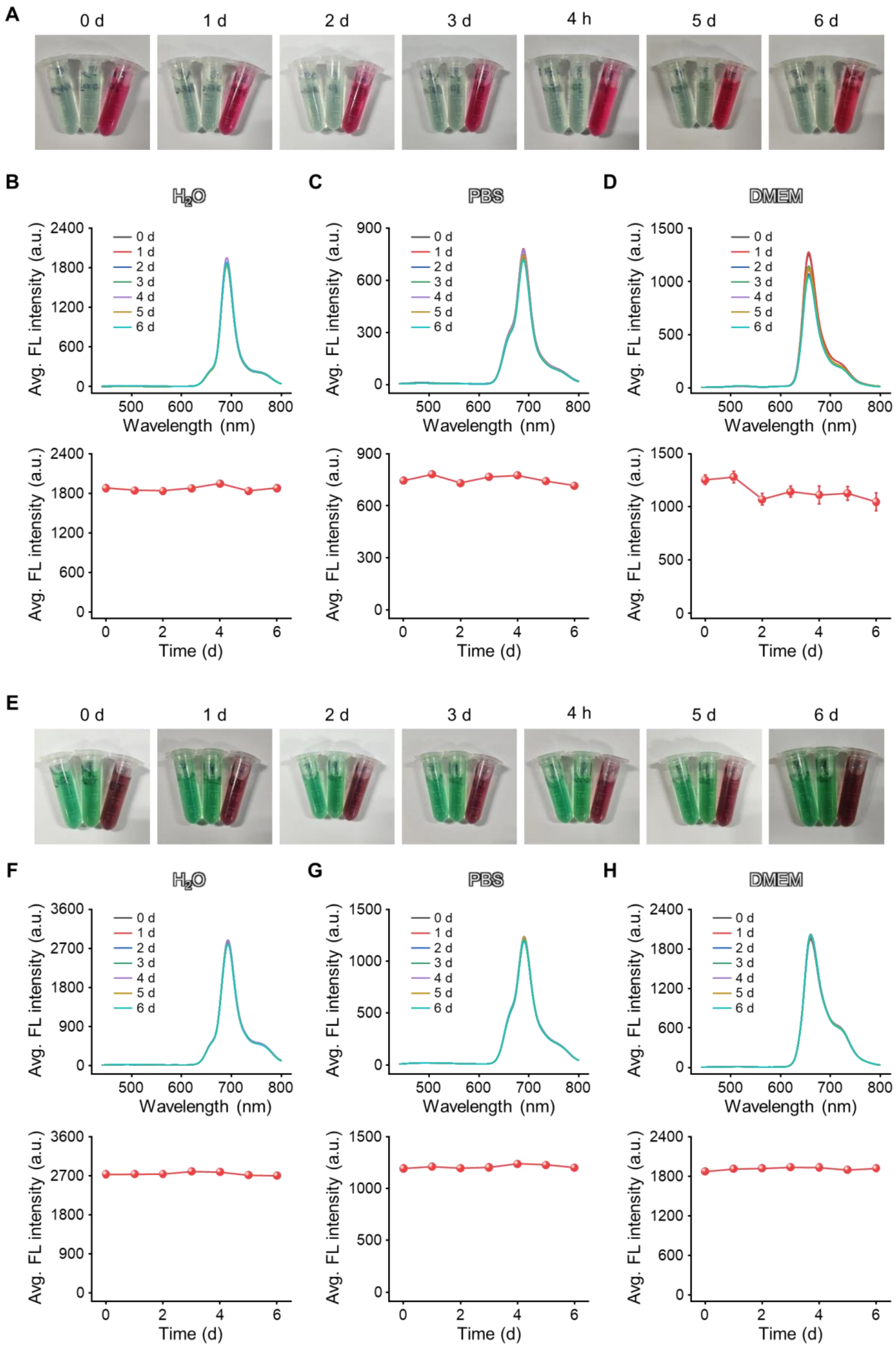

Figure S7. A) Digital photographs of 10 μg/mL pep-CDs in different media during the whole observation period from 0 to 6 d (from left to right are H_2O, PBS and DMEM.). B-D) FL emission spectra (λ_{ex} = 420 nm) and stability of 10 μg/mL

pep-CDs in different media during the whole observation period from 0 to 6 d. E) Digital photographs of 50 μg/mL pep-CDs in different media during the whole observation period from 0 to 6 d (from left to right are H_2O, PBS and DMEM.). F-H) FL emission spectra (λ_{ex} = 420 nm) and stability of 50 μg/mL pep-CDs in different media during the whole observation period from 0 to 6 d. Data are illustrated as mean ± s.d. (n = 3).

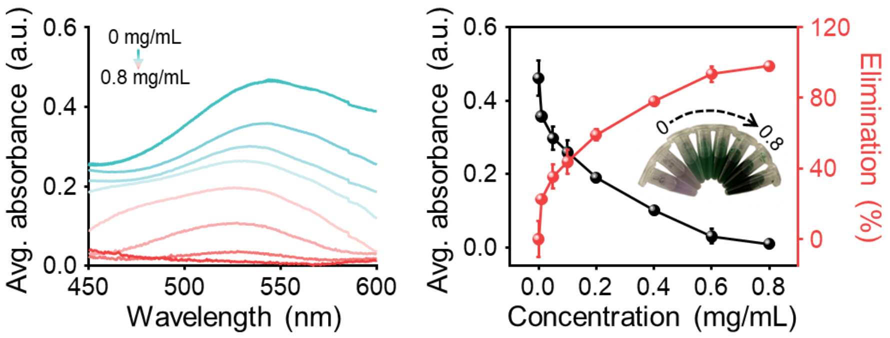

Figure S8. UV-Vis absorption spectra of $DPPH^{\bullet}$ after incubation with different concentrations of CDs, and the corresponding absorbance of each system and the elimination efficiency of $DPPH^{\bullet}$ by CDs. Data were illustrated as mean ± s.d. (n = 3).

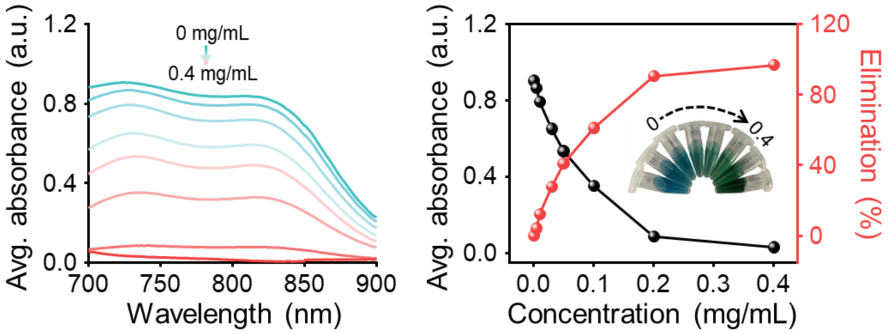

Figure S9. UV-Vis absorption spectra of $ABTS^{\bullet+}$ after incubation with different concentrations of CDs, and the corresponding absorbance of each system and the elimination efficiency of $ABTS^{\bullet+}$ by CDs. Data were illustrated as mean ± s.d. (n = 3).

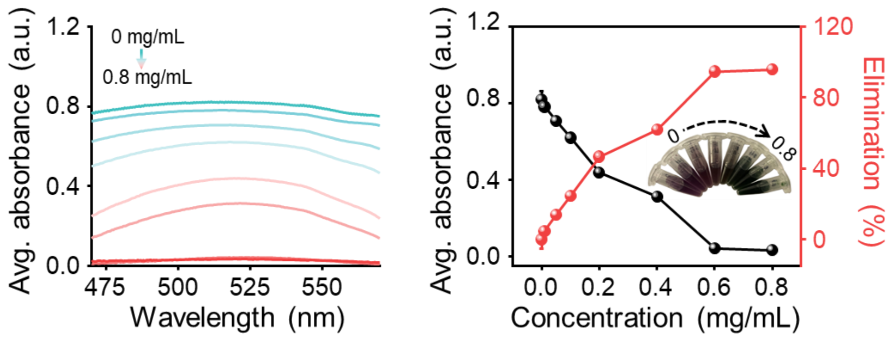

Figure S10. UV-Vis absorption spectra of $^{\bullet}OH$ after incubation with different concentrations of CDs, and the corresponding absorbance of each system and the elimination efficiency of $^{\bullet}OH$ by CDs. Data were illustrated as mean ± s.d. (*n* = 3).

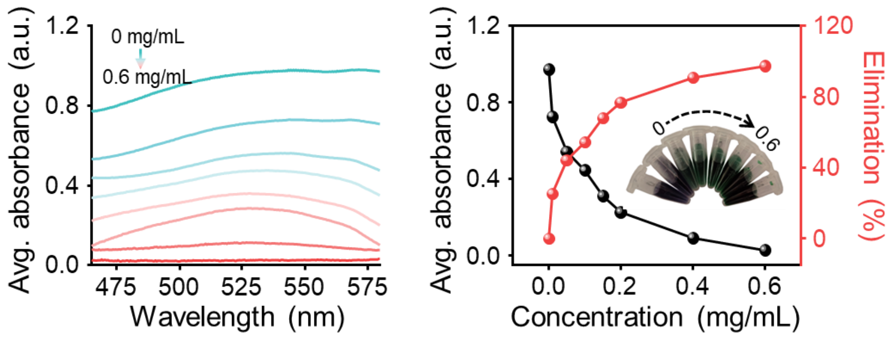

Figure S11. UV-Vis absorption spectra of $O_2^{\bullet -}$ after incubation with different concentrations of CDs, and the corresponding absorbance of each system and the elimination efficiency of $O_2^{\bullet -}$ by CDs. Data were illustrated as mean ± s.d. (*n* = 3).

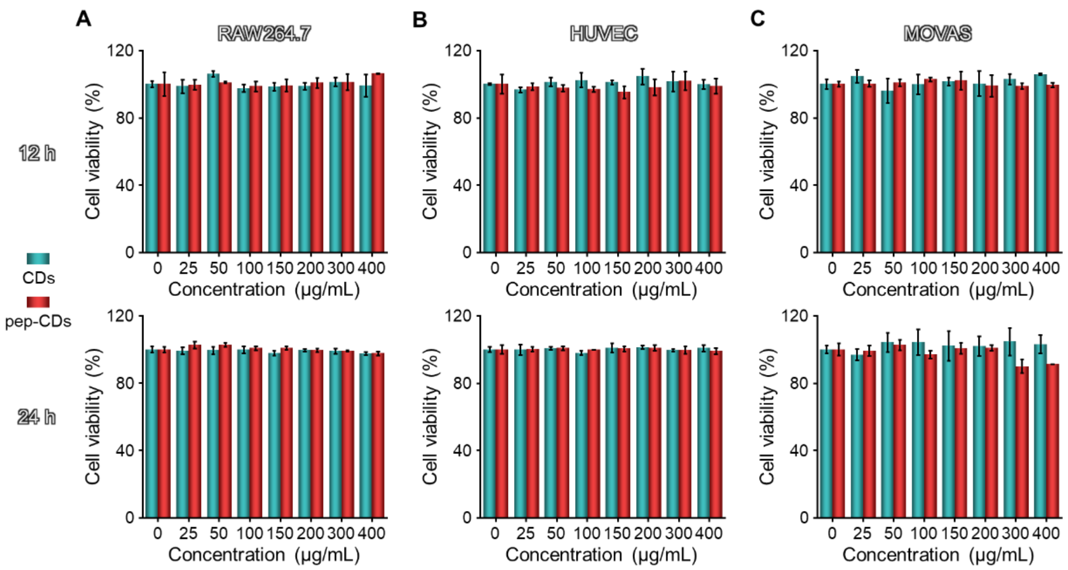

Figure S12. Cell viability of A) RAW264.7, B) HUVEC, and C) MOVAS cells after treatment with various concentrations of CDs and pep-CDs (from 0 to 400 μg/mL) for 12 and 24 h. Data were illustrated as mean ± s.d. (n = 3).

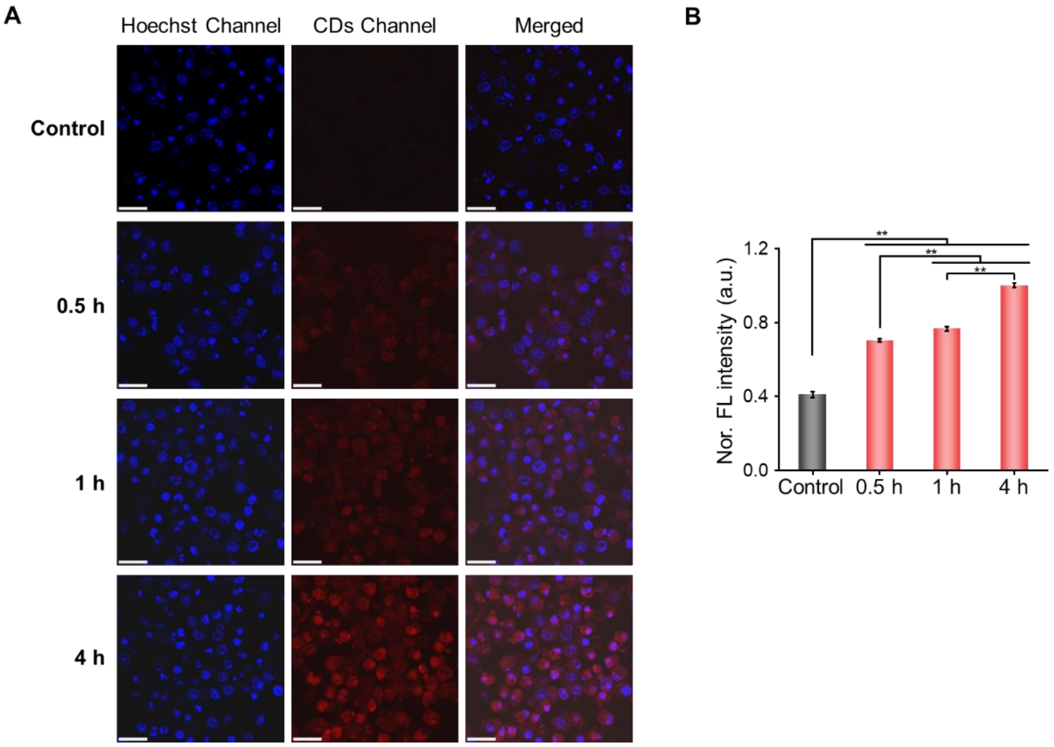

Figure S13. A) CLSM images and B) corresponding quantitative analysis of FL signal intensities of RAW264.7 macrophages pre-incubated with ox-LDL for 48 h and

then treated with pep-CDs for various times (scale bars: 25 μm). Data were illustrated as mean ± s.d. (n = 3). **p < 0.01.

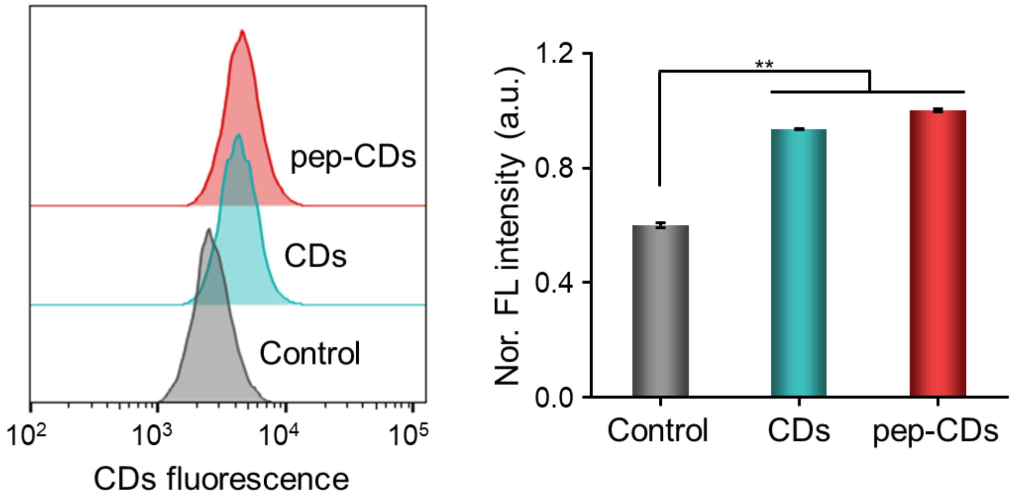

Figure S14. The flow cytometry results and quantification of cellular uptake of CDs and pep-CDs in RAW264.7 cells. Data were illustrated as mean ± s.d. (n = 3). **p < 0.01.

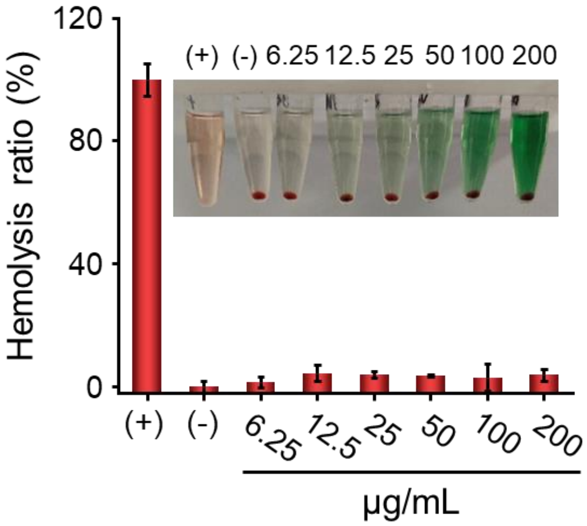

Figure S15. Digital photos and hemolysis rates of mice blood processed with water (positive control (+)), PBS (negative control (-)), and pep-CDs at various

concentrations (from 6.25 to 200 μg/mL). Data were illustrated as mean ± s.d. (n = 3).

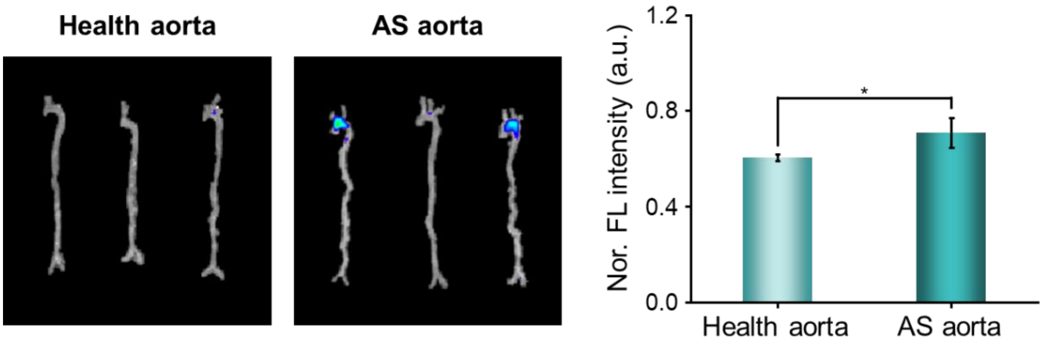

Figure S16. *Ex vivo* FL images and corresponding quantitative analysis of FL signal intensities of health and AS aortas from C57 and ApoE$^{-/-}$ mice fed on HFD for 10 w at 2 h post CDs *i.v.* injection. Data were illustrated as mean ± s.d. (n = 3). *p < 0.05,.

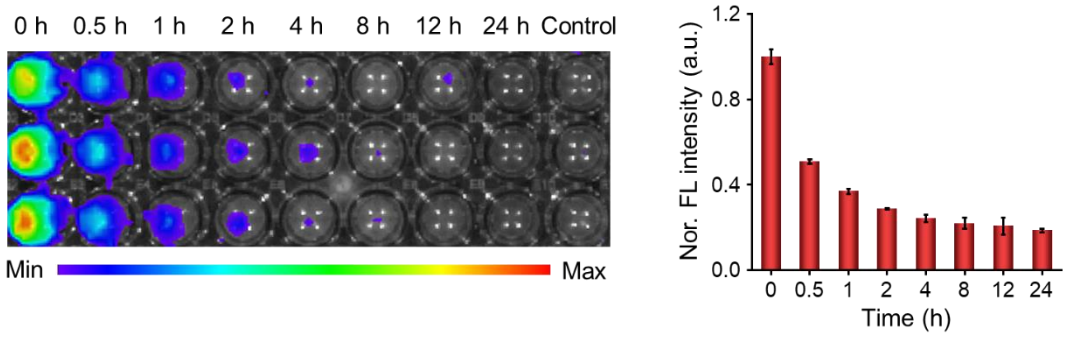

Figure S17. *In vivo* pharmacokinetic performance of pep-CDs after *i.v.* injection in mice. Data were illustrated as mean ± s.d. (n = 3).

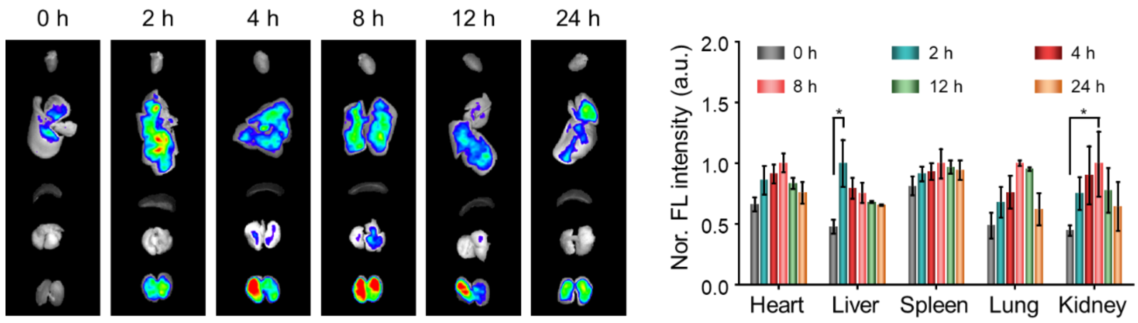

Figure S18. FL images and corresponding quantitative analysis of FL signal intensities of the major organs (heart, liver, spleen, lung, and kidney) from ApoE$^{-/-}$ mice fed on HFD for 10 w at various time points after *i.v.* injection of pep-CDs. Data were illustrated as mean ± s.d. ($n = 3$). $*p < 0.05$.

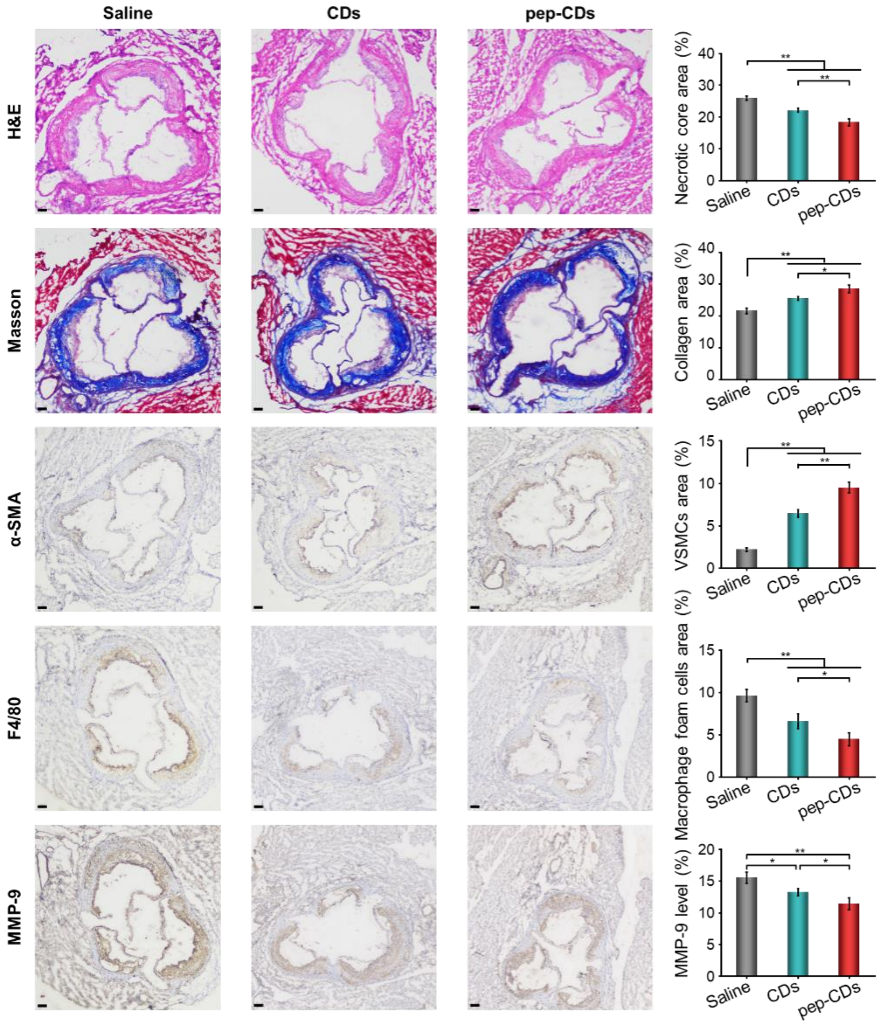

Figure S19. H&E, Masson's trichrome, and α-SMA, F4/80, and MMP-9 immunohistochemistry staining images and corresponding quantitative analysis of the plaque necrotic core, plaque collagen area, plaque VSMCs area, plaque macrophage foam cells area, and plaque MMP-9 expression levels of aortic root cryosections from ApoE$^{-/-}$ mice after different treatments (scale bars: 100 μm). Data were illustrated as mean ± s.d. (n = 4-5). *p < 0.05, **p < 0.01.

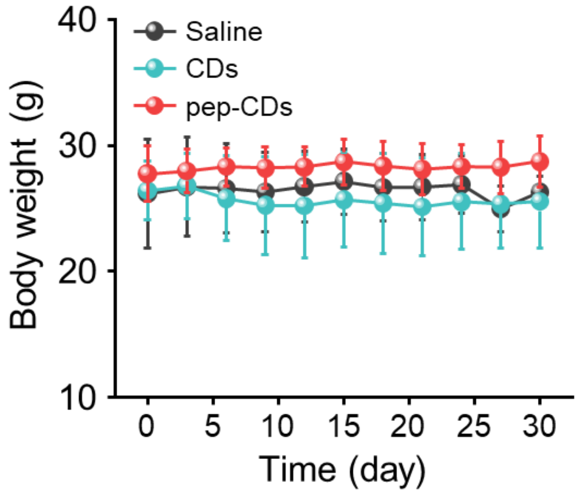

Figure S20. Body weight variation curves of ApoE$^{-/-}$ mice from different treatment groups. Data were illustrated as mean ± s.d. (*n* =4-5).

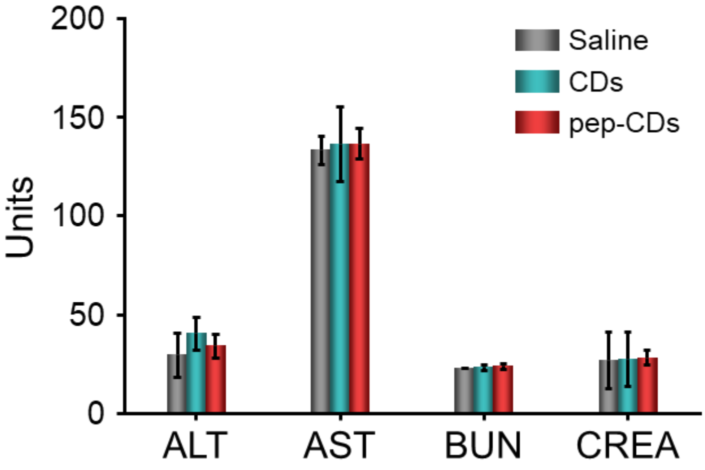

Figure S21. Levels of biochemical markers relevant to hepatic and kidney functions in serum. ALT, alanine aminotransferase; AST, aspartate aminotransferase; BUN, blood urea nitrogen; CREA, serum creatinine. Data were illustrated as mean ± s.d. (*n* = 4-5).

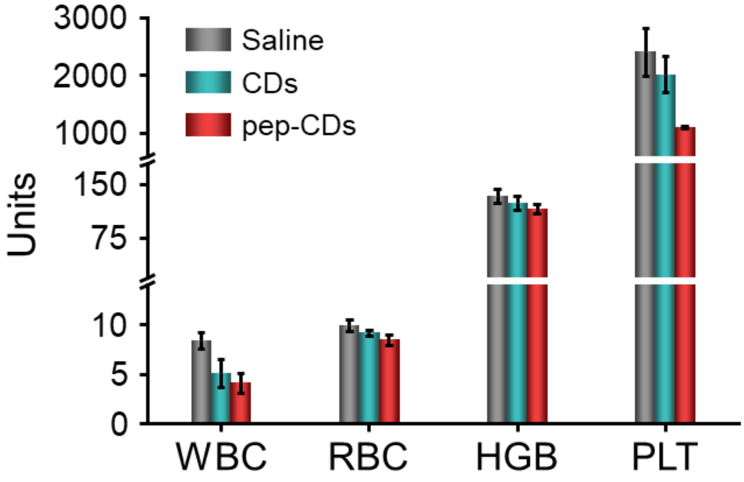

Figure S22. Levels of typical hematological parameters in serum. WBC, white blood cell; RBC, red blood cell; HGB, hemoglobin; PLT, platelet. Data were illustrated as mean ± s.d. (n = 4-5).

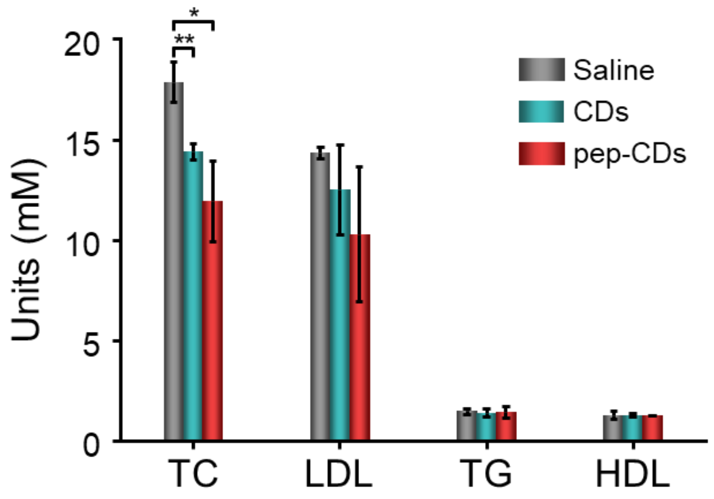

Figure S23. Levels of typical lipid markers in serum. TC, total cholesterol; LDL, low-density lipoprotein; TG, triglyceride; HDL, high-density lipoprotein. Data were illustrated as mean ± s.d. (n = 4-5). *$p < 0.05$, **$p < 0.01$.

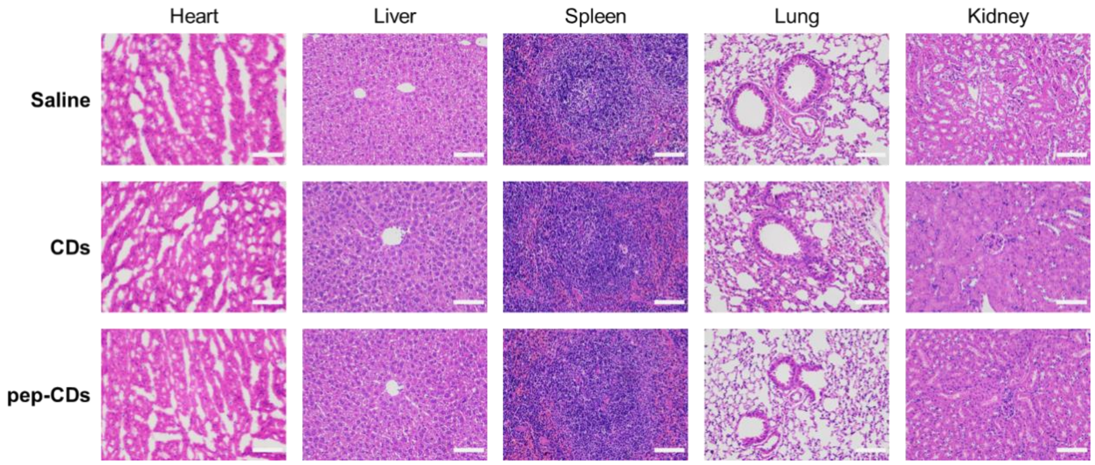

Figure S24. H&E staining of major organs sections from ApoE$^{-/-}$ mice after different treatments. All scale bars: 100 μm.
